# Excitonic Configuration Interaction: Going Beyond the Frenkel Exciton Model

**DOI:** 10.1021/acs.jctc.4c00157

**Published:** 2024-06-17

**Authors:** Tomislav Piteša, Severin Polonius, Leticia González, Sebastian Mai

**Affiliations:** † Institute of Theoretical Chemistry, Faculty of Chemistry, University of Vienna, Währinger Straße 17, Vienna 1090, Austria; ‡ Vienna Doctoral School in Chemistry (DoSChem), University of Vienna, Währinger Str. 42, Vienna 1090, Austria; § Vienna Research Platform Accelerating Photoreaction Discovery, University of Vienna, Währinger Straße 17, Vienna 1090, Austria

## Abstract

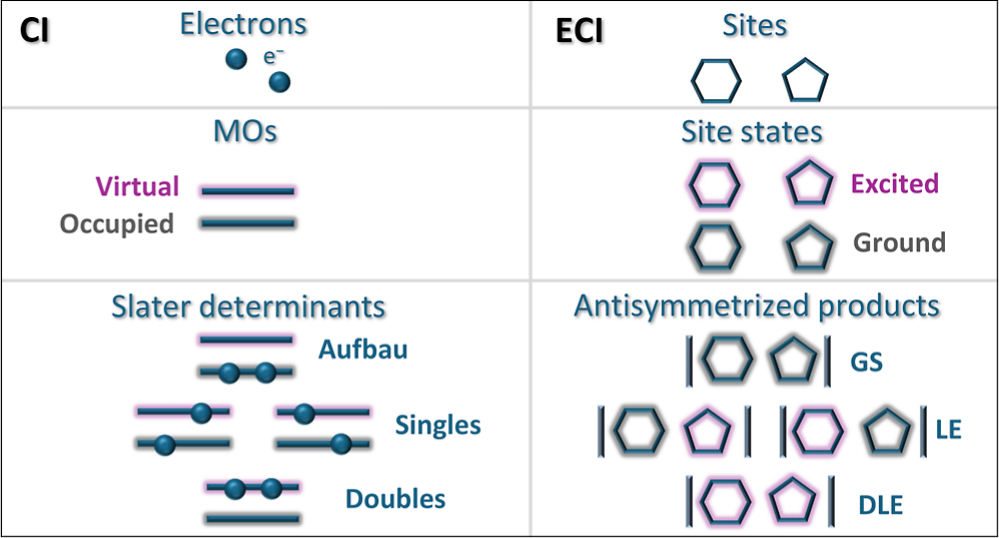

We present the excitonic configuration interaction (ECI) method—a fragment-based analogue of the CI method for electronic structure calculations of multichromophoric systems. It can also be viewed as a generalization of the exciton approach, with the following properties: (i) It constructs the effective Hamiltonian exclusively from monomer calculations. (ii) It employs the strong orthogonality assumption and is exact within McWeeny’s group function theory, thus requiring only one-electron density matrices of the monomer states. (iii) It is agnostic of the monomer electronic structure method, allowing us to use/combine different methods. (iv) It includes an embedding point charge scheme (called excitonic Hartree–Fock, EHF) to improve the accuracy of the monomer states, but such that the effective full-system Hamiltonian is not explicitly dependent on the embedding. (v) It is systematically improvable, by expanding the set of monomer states and by including configurations where two or more monomers are excited (defining the ECIS, ECISD, etc., methods). The performance of ECI is assessed by computing the absorption spectrum of two exemplary multichromophoric systems, using CIS as the monomer electronic structure method. The accuracy of ECI significantly depends on the chosen embedding charges and the ECI expansion. The most accurate assessed combinations—ECIS or ECISD with EHF embedding—yield spectra that agree qualitatively and quantitatively with full-system direct calculations, with deviations of the excitation energies below 0.1 eV. We also show that ECISD based on CIS monomer calculations can predict states where two monomers are excited simultaneously (e.g., triplet–triplet double-local excitations) that are inaccessible in a full-system CIS calculation.

## 1 Introduction

The accurate description of electronic excited states is of central importance to simulate electronic spectra and photoinduced molecular dynamics in molecules. Nowadays, a large array of electronic structure methods for such calculations is available,^1^ ranging from single-^2^ to the multireference^3^ methods. However, all of those methods scale unfavorably with system size, which hinders their applicability to large structures. Particularly challenging are multichromophoric systems—assemblies consisting of different moieties (typically called chromophores, fragments, or sites, which we use interchangeably in this article) that each contribute low-lying (localized) excited states to the system. These localized states can interact with each other and form “excitonic states”, linear combinations of localized excitations.^4,5^ Such multichromophoric systems are ubiquitously found in nature, for example, DNA^6^ or the light-harvesting complexes of the chloroplasts,^7,8^ and also have attracted significant attention in the field of material design, e.g., in photovoltaics^9^ or organic semiconductors.^10^ Hence, understanding their photophysical and photochemical properties has become a top priority in modern spectroscopy. Unfortunately, if the spectra of different chromophores are overlapping, multichromophoric systems can acquire a very large density of states at low energies, making their electronic structure very challenging to describe. For the same reason, the ultrafast excitation energy transfer taking place in such systems after local photoexcitation^11^ is very hard to simulate with excited-state molecular dynamics.

One alternative to the use of excited-state electronic structure methods to treat the full multichromophoric system at once is to use exciton models,^12,13^ which can be considered a special case of the very large class of fragment-based electronic structure methods.^14,15^ The idea behind exciton models is to first compute the ground and excited states of individual chromophores separately (obtaining the so-called site states) and then to construct the full-system model Hamiltonian matrix in the basis of the antisymmetrized products of the fragments’ states (the so-called excitonic basis). After the model Hamiltonian matrix is obtained, its diagonalization provides the full-system states. In this way, local correlation and excitation is taken care of in the site-state calculations, and the typically weaker interfragment interactions are treated at the full-system level.

Several such fragment-based excited-state approaches have been devised, differing significantly in their level of sophistication. Many practically employed exciton models^16−19^ apply several approximations, related either to the truncation of the basis of the antisymmetrized products or to approximations used in evaluating the matrix elements. Other previously proposed fragment-based methods with a similar philosophy evaluate the Hamiltonian matrix elements in principle exactly, e.g., excitonic coupled cluster,^20,21^ the localized active space-state interaction method,^22^ or the active space decomposition method.^23,24^ Such variational “exact matrix element” framework can also be implemented by other general (not necessarily fragment-based) approaches, such as the generalized active space methods,^25^ and allow studying multifragment systems with very strongly correlated fragments. However, if the chromophores are not particularly close to each other, one can assume that the antisymmetrized products are strongly orthogonal. This is called the strong orthogonality assumption and leads to much simpler and computationally less demanding expressions for the Hamiltonian matrix elements, and hence, many exciton models rely on it. The corresponding expressions were obtained by McWeeny in his group function theory (GFT)^26^ and were used to study intermolecular interactions.

The Frenkel exciton model (FEM),^4^ later extended by Davydov to molecular multichromophoric systems,^5,27^ is arguably the most well known of the exciton models. It employs an excitonic basis that consists only of the product of the site ground states (the so-called GS product) and all the products with exactly one excited site state, referred to as the local excitation (LE) products. The expressions for the matrix elements in FEM, although not historically obtained like that, actually correspond to the GFT expressions with some additional approximations (as will be recapitulated below).

Despite numerous approximations, the original FEM appears to perform quite well for systems with weakly interacting chromophores, such as molecular aggregates.^27^ However, driven by the need to describe multichromophoric systems with more strongly interacting fragments and encompassing states without LE character, many improvements of the FEM emerged in the past decade,^16−19,28,29^ each of them lifting a particular approximation in a certain way. GFT, FEM, and some of the recent extensions of the FEM will be presented in more detail in Section 2, which reviews the mathematical background used in this work.

In this work, we present an excitonic method, denoted as excitonic configuration interaction (ECI). The main features of this methods are the following. (i) Its effective Hamiltonian is constructed exclusively from monomeric site states, i.e., no dimer or oligomer calculations are required. This should give the method near linear scaling with the number of fragments. (ii) It is exact within GFT and employs the strong orthogonality assumption, which guarantees that the Hamiltonian can be constructed in a cheap manner only from site-state energies and one-electron (transition) densities.^26^ (iii) It is constructed in a way nearly agnostic of the electronic structure method used to calculate the site states, and in principle even allows using different levels of theory for different fragments. (iv) It uses an embedding point charge scheme to optimize the site states, but in such a way that the full-system energetics does not explicitly depend on the embedding charges. In other words, it should be systematically improvable to the same results independent of the employed embedding charges, while the usage of the more favorable charges only accelerates the convergence of the method with the number of the site states.

In more detail, ECI employs an excitonic basis comprising not only GS and LE products but also multilocal excitation (MLE) products, i.e., those with more than one excited site state. Furthermore, it treats the ground and the excited full-system states on an equal footing, which is not characteristic for the exciton models. For these reasons, ECI is a generalization of the exciton approach, which is why we call it an excitonic (or exciton-like) method rather than an exciton method. As said, the ECI Hamiltonian is exact within GFT, which makes ECI somewhat more expensive than the FEM, but—as the evaluation of the GFT formulas is rather cheap—still significantly cheaper than “exact matrix element” methods like the ones mentioned above.^22−24^ Hence, ECI can be viewed as a bridge between exciton models and exact fragment-based methods. We also propose a modification of the GFT formulas so that the site states can be calculated with embedding via arbitrary point charges placed on the positions of the nuclei of the other chromophores. We show that these charges can be varied to optimize the energy of the full-system ground state and used in the calculation of the excited site states. Further, we propose a simple algorithm to exclude the site states that might lead to the violation of the strong orthogonality assumption and show that the GFT-based approach can be used even for systems with strong interactions between chromophores. The entire ECI methodology will be presented in detail in Section 3.

**Figure 1:**
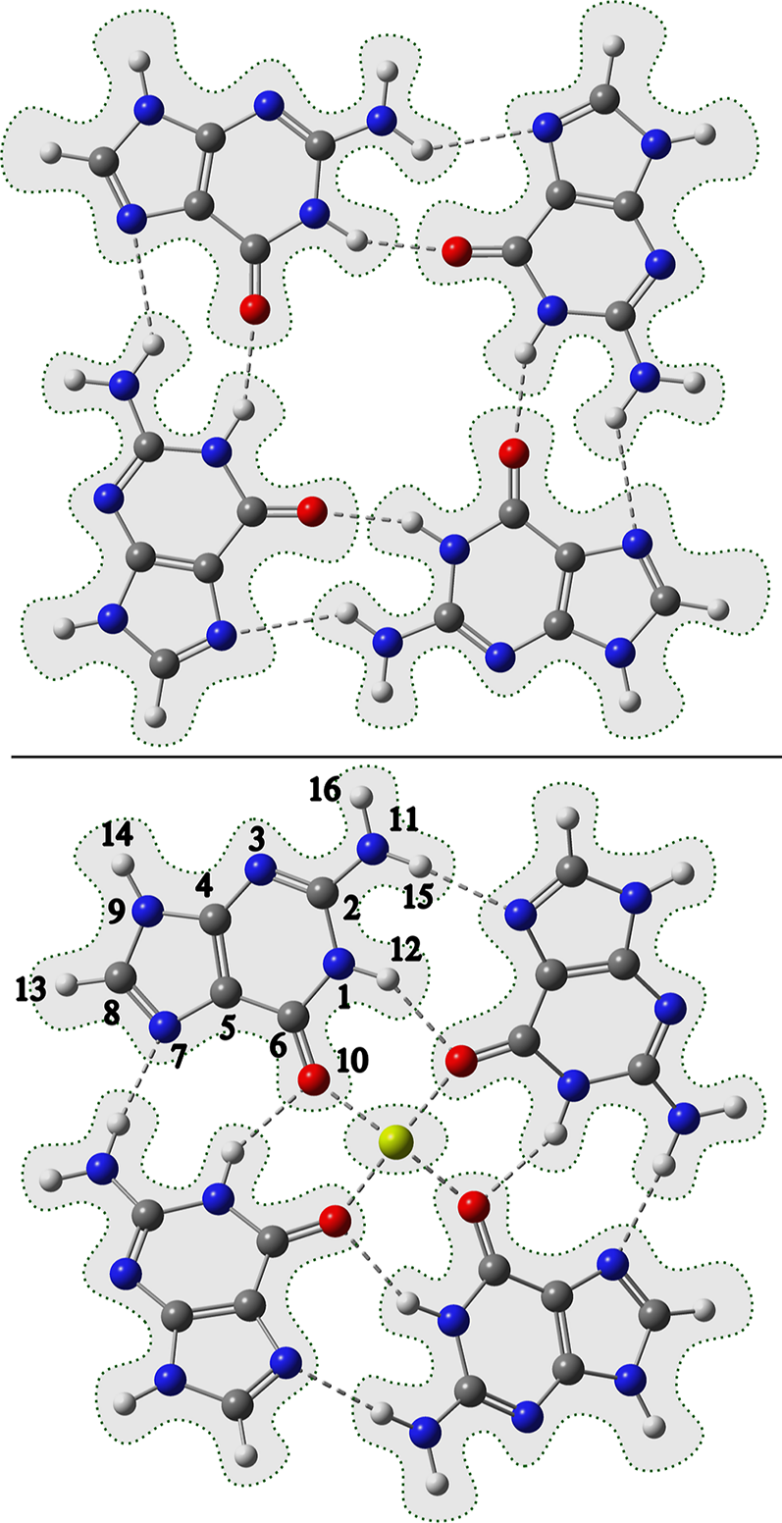
Molecular structures of **G**_**4**_ (top) and **MgG_4_^2+^** (bottom) with fragments emphasized by the dotted shaded frames. The numeration of the guanine atoms is given on one guanine moiety in **MgG_4_^2+^**.

The performance of the ECI method for the calculation of electronic absorption spectra is illustrated on the two model multichromophoric systems shown in Figure 1. The first one is a single layer of the guanine-quadruplex (**G**_**4**_), exhibiting double hydrogen bonds between guanine moieties connected in the supramolecular ring. This motif is often found in the telomeric regions of DNA and is often stabilized by a metal cation.^30^ Hence, the second test system is **G**_**4**_ with a Mg^2+^ cation in the center (**MgG_4_^2+^**), which constitutes the more challenging test due to the presence of metal–ligand bonds and of stronger cross-fragment polarization. The **G**_**4**_ system was treated as a four-fragment system (each guanine as a single fragment) and the **MgG_4_^2+^** system as a five-fragment system, with the Mg^2+^ cation being one fragment for itself (computational details in Section 4). The performance of ECI on these two systems will be presented and discussed in Section 5.

## 2 Theoretical Background

Let us consider a multichromophoric system consisting of *M* fragments, labeled *F* = 1, ..., *M*. The nonrelativistic electronic Hamiltonian of the system can be written as
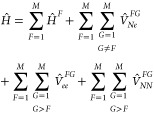
1where  is the electronic Hamiltonian of fragment *F*. The operators , , and  describe the potential energy of nuclear–electronic, electronic–electronic, and nuclear–nuclear interaction between the respective particles of fragment *F* and fragment *G*, respectively.

Let each chromophore have a set of site states
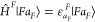
2which are labeled by two numbers, the first one denoting the fragment (*F*) and the second one denoting the site state (*a*_*F*_), where *a*_*F*_ = 0 corresponds to the site’s ground state. The eigenvalues of the site Hamiltonians  are usually referred to as the site energies. With this notation, a member function of the excitonic basis can be written as
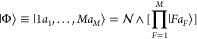
3where  is the normalization constant and ∧ is the partial antisymmetrizer, acting on a product of site states and making it antisymmetric to the permutation of two electrons on two different fragments. As the site states are already antisymmetric to the permutation of electrons within the site, the |Φ⟩ are thus antisymmetric to the permutation of any two electrons in the system.

### 2.1 Group Function Theory

This subsection presents the expressions of the matrix elements of *Ĥ* in the excitonic basis within the strong orthogonality assumption. The expressions were first presented in 1959^31^ and more recently collected in ref (26) (eqs 14.1.7–14.1.10, 14.1.13, and 14.3.10 within).

The excitonic basis is said to be strongly orthogonal if for any two site-state products Φ and Θ
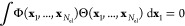
4holds for any possible values of **x**_2_,...,**x**_*N*_el__, where *N*_el_ is the total number of electrons in the system. As will be shown, the GFT expressions obtained under this assumption can be evaluated entirely from the site energies and site-specific 1-particle density matrices and hence are much cheaper to evaluate than the exact matrix elements. Within the strong orthogonality assumption, the normalization constant for each antisymmetrized product reads
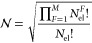
5where *N*_el_^*F*^ is the number of electrons of fragment *F*.

Before presenting the GFT expressions, we define the two-fragment Coulomb (*J*) and exchange (*K*) terms as
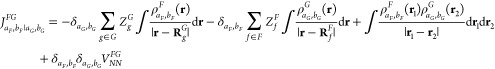
6and
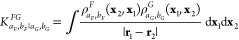
7In these definitions, *F* and *G* denote the indices of two fragments; *a*_*F*_, *b*_*F*_, *a*_*G*_, and *b*_*G*_ denote the indices of site states of the corresponding fragment; and δ_*a*,*b*_ are Kronecker deltas. If *a*_*F*_ = *b*_*F*_, then  is the electron density of a site state, while if *a*_*F*_ ≠ *b*_*F*_, it is a transition density between two site states. The quantity  is the corresponding 1-particle (state or transition) density matrix, nonintegrated over spin. Furthermore, the scalar *V*_*NN*_^*FG*^ is the constant interaction energy between the nuclei of fragments *F* and *G*, **R**_*g*_^*G*^ is the position of the *g*-th nucleus of fragment *G*, and *Z*_*g*_^*G*^ is its charge. Note that the *J* terms can simply be integrated over the spin coordinates, regardless of the multiplicities of the involved site states. For the *K* terms, integration over the spin coordinates produces a more complex expression involving partial density matrices, as given in eqs 47 and 49 in Appendix B.

With these definitions, the diagonal matrix element ⟨Φ|*Ĥ*|Φ⟩, where Φ is given in eq 3 within GFT, is given by (dropping out the explicit limits in summations)
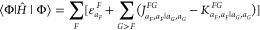
8For the off-diagonal elements, we distinguish three cases. The coupling between Φ and —where the latter product differs in only one site state with respect to the former one (containing *Fb*_*F*_ instead of *Fa*_*F*_)—reads
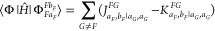
9If the products in bra and ket differ in exactly two site states, corresponding to fragments *F* and *G*, the matrix element is
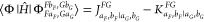
10If bra and ket products differ in more than two site states, the matrix element is zero.

As can be seen, McWeeny’s GFT formulas are fully analogous to the Slater–Condon expressions for the matrix elements of the electronic Hamiltonian in the basis of Slater determinants, with fragments playing the role of electrons and site states playing the role of molecular orbitals. If the bra and ket products are equal, the matrix element includes the sum of site energies (instead of the sum of orbital energies), interfragment nuclear–electron interaction integrals (instead of 1-electron integrals), and interfragment electron–electron interaction and exchange integrals (instead of 2-electron integrals). If the bra and ket products differ in one site state, the coupling contains nuclear–electron and electron–electron integrals. If they differ in two site states, the coupling includes only electron–electron integrals. The Kronecker deltas in the *J* term (eq 6) serve as switches to include the respective terms depending on the number of differing site states. Note, however, that the aforementioned analogy between GFT and regular electronic structure theory does not hold perfectly—the fragments are distinguishable, whereas electrons are indistinguishable, and the site states are not one- but rather multifermionic functions themselves.

Following the logic of the GFT, McWeeny furthermore developed (Chapter 14.2 of ref (26)) an approach for the variational calculation of the full system using a single antisymmetrized product given in eq 3 as the test function, and varying each site state in order to minimize the expected value of energy, given by eq 8. This simplifies to the iterative minimization of each summand in eq 8 (one for each chromophore *F*) separately, until the self-consistency is reached, i.e., until all site states stop changing. If one starts from the antisymmetrized product of the site ground states, the obtained state will presumably be an estimation of the full-system ground state, and this procedure can be viewed as the GFT analogue of the Hartree–Fock (HF) method.

### 2.2 Frenkel Exciton Model

As mentioned above, the excitonic basis in the FEM contains only GS and LE products, which can be written in the present notation as |**0**⟩ = |10,...,*M*0⟩ and , respectively. Just like in the previous section,  denotes that the LE product is obtained from the GS product by exciting the site *F* from site state *F*0 to *Fa*_*F*_. In the following, we will occasionally drop out the symbol of the site ground state in the subscript (i.e., we write  instead of ) to simplify the notation. The excitonic basis in the FEM is not complete, as the complete excitonic basis should also contain all MLE products, and additionally charge-transfer (CT) products. The latter are products which include cationic and anionic site states (relative to some reference charge distribution per site), but having the same total charge as the GS, LE, and MLE products.

The matrix elements of the full-system Hamiltonian in the FEM are given by the GFT expressions (8) to (10), but with the *J* and *K* terms in expressions (8) and (9) neglected. In other words, the diagonal matrix elements in the FEM are just the sums of site energies, and the diagonal cross-fragment interaction contribution is neglected. Further, the GS–LE couplings, , and the couplings between two LEs on the same site,  (*a*_*F*_ ≠ *b*_*F*_), are fully neglected from eq 9 in GFT. The only considered couplings in the FEM are those between LEs on two different sites, , which are given by eq 10.

With all of these simplifications, the Hamiltonian of the FEM becomes
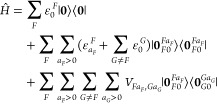
11As can be seen, it has only two sets of parameters—the site energies  and the couplings between LEs on the different sites (*V*_*Fa*_*F*_,*Ga*_*G*__). While the former are directly obtained from the site-state calculations, the latter can be subsequently calculated by eq 10, i.e., as
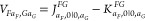
12If the chromophores are relatively far apart, the *K* term in eq 12 is often also neglected, which is equivalent to the usage of Hartree products of site states instead of antisymmetrized products of site states.

Another issue of the original FEM is the fact that the site states are obtained by a series of electronic structure calculations of the *isolated* sites, which is often referred to as the frozen density approximation (FDA).^18^ This actually would not be an approximation, if the complete set of site states was provided on each chromophore and if a complete excitonic basis was employed. However, because the first requirement is impossible in practice (one always has a finite number of site states), and since the second requirement is not the case in the FEM (the Frenkel basis contains only GS and LE products), in practice the FDA is an approximation within the FEM. Thus, the FDA can significantly affect the performance of the FEM, especially if the fragments are close and strongly interacting. This is because the electronic wave functions of the isolated sites do not necessarily resemble the wave function of the full system in the vicinity of the respective sites due to the perturbation one fragment causes to the other. Therefore, if the FDA is employed, one would have to provide a very large set of site states on each site in order to recover the deformations of the wave functions that happen when the chromophores are joined to the full system.

As mentioned above, some of the approximations of the FEM were lifted in different FEM-like models developed in the past decade. For example, Menger et al.^16^ and Sangiogo Gil et al.^17^ developed FEM-based exciton models in which the FDA was mitigated by means of QM/MM schemes based on electrostatic embedding. In these models, the site states on a single fragment are obtained by an electronic structure calculation of that fragment in the presence of all other fragments represented as point charges, where the latter are obtained via some molecular mechanics force field. The inclusion of the force-field charges perturbs the site states of the considered fragment, approximately like the other sites in their ground states would do in the full system. The excitonic basis obtained from these perturbed site states is expected to be variationally more favorable than a basis generated from FDA-based site states in the original FEM. Furthermore, the QM/MM approach enables those models to approximately account for the cross-fragment interaction energy on the diagonal (see the *J* and *K* terms in eq 8), although under the assumptions that MM fragments are always in the ground state and that parts of the interaction energy are evaluated using additional MM calculations.^16,17^ In the mentioned two models, all other approximations of FEM were inherited. Nonetheless, both models have been already used in surface hopping simulations of the nonadiabatic dynamics of multichromophoric systems, showing promising performance.^16,17^ To be mentioned, exciton approach has been shown generally applicable for the simulation of the light-induced molecular dynamics.^32,33^ Regarding the embedding-improved FEM-like models, we emphasize the model of Morrison et al.,^28^ in which a self-consistent point-charge embedding scheme has been used in the site-state calculations, based on the so-called XPol approach.^34^ This is supposed to be more sophisticated point-charge embedding than the QM/MM electrostatic embedding, as it takes into account the self-consistency between chromophores. Further, some other works extend the FEM by including CT products in the excitonic basis,^18,19,35^ although we note that Li et al.^18^ neglected the GS–LE and GS–CT couplings, stressing that these would break the size extensivity of the model. Finally, there are improvements^27,28^ of the FEM in which the nonorthogonality between the antisymmetrized products is also taken into account, going beyond the GFT framework, but inheriting the other simplifications of the FEM (see Section 3.2.1).

## 3 Theory

### 3.1 Embedding via Arbitrary Point Charges

Adopting the notation used in Section 2, we add and subtract “embedding interaction” terms to the electronic Hamiltonian of a multichromophoric system, which gives
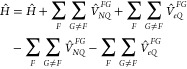
13where the operators  and  describe the interaction energy between nuclei (*N*) and electrons (*e*) of fragment *F* with embedding charges *Q* placed at the nuclear positions of fragment *G*. As *Ĥ* does not explicitly depend on these charges (due to adding and then subtracting the interaction terms), these charges can in principle be chosen arbitrarily, without changing *Ĥ* and the exact eigenstates of *Ĥ*.

It is convenient to rearrange the expression as (note the tildes)
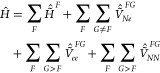
14where
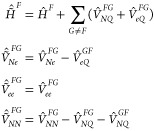
15Now, let each  have its eigenstates
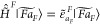
16Those states can be obtained by an electronic structure calculation of fragment *F* with the embedding charges of all other fragments *G* included as external point charges (i.e., like in electrostatic embedding). Clearly,  and  parametrically depend on the embedding charges of all other fragments. In the following, we will refer to all quantities related to those states as “embedded” quantities (e.g.,  is the embedded site Hamiltonian,  is an embedded site state, and  is an embedded site energy), denoted by the tildes.

### 3.2 Excitonic Configuration Interaction

Throughout the rest of the article, *S* and *M*_*S*_ represent the quantum number of the spin and its *z*-component of the full-system wave functions (e.g., antisymmetrized products or the full-system states), while the small-case *s*^*F*^ and *m*_*s*_^*F*^ represent the corresponding quantum numbers of the site states.

#### 3.2.1 ECI Basis and ECI Hamiltonian

We turn to the excitonic variational calculation of the full-system states in the basis of the antisymmetrized products of the embedded site states (i.e., embedded products)
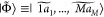
17while only assuming that the products are strongly orthogonal, as in GFT. For this calculation, we need to find the expressions for the matrix elements , in order to build the full-system Hamiltonian matrix in the basis of embedded products. We do this simply by noticing that the two forms of the full-system Hamiltonian, given in eqs 1 and 14, are of the same shape. This implies that the expressions for  have to be of the same shape as the GFT expressions (8), (9), and (10) for ⟨Θ|*H|*Φ⟩. Considering the relationships given in expression (15), one sees that only the following modifications
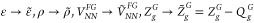
18have to be applied to the GFT formulas 8–10, where *Q*_*g*_^*G*^ is the embedding charge of the *g*-th nucleus of fragment *G*. These modified GFT formulas inherit the analogy of the original GFT formulas with the Slater–Condon rules. Hence, we refer to such a variational calculation of the full-system states as “excitonic configuration interaction” (ECI) and to the embedded products as “excitonic Slater determinants” (ESDs). Also, for the sake of brevity, we will refer to to the GFT formulas 8–10 with modifications (18) as the “ECI formulas”. Not to be mistaken by the name, ESDs are not *N*_el_ × *N*_el_ Slater determinants, but rather linear combinations of *N*_el_ × *N*_el_ Slater determinants, because the embedded site states are linear combinations of the site-specific (i.e., *N*_el_^*F*^ × *N*_el_^*F*^) Slater determinants.

**Figure 2:**
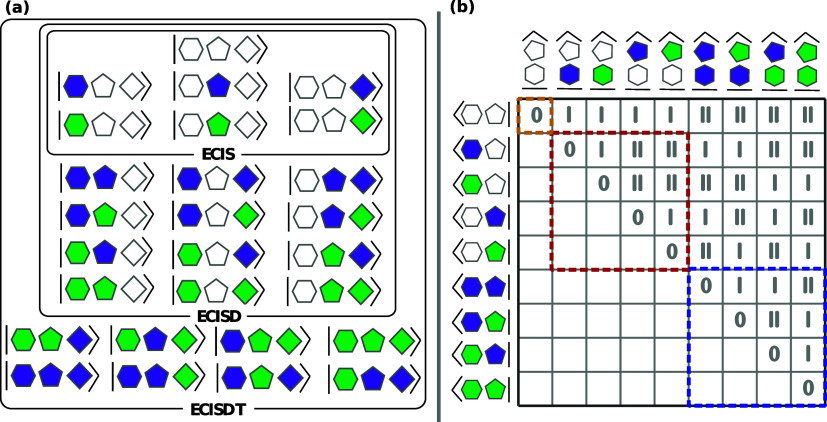
(a) Schematic representation of the excitonic basis of ECIS, ECISD, and ECISDT bases on an example of a trichromophoric system with three site states (ground and two excited) supplied on each fragment. The chromophores are represented by hexagon, pentagon, and square, while the site states are denoted by color (white: ground site state, purple and green: two excited site states). ESDs are then shown as schematic kets containing the site states. (b) Schematic representation of the full-system Hamiltonian matrix in the ECI basis of a bichromophoric system (hexagon and pentagon) with three site states (ground and two excited states) supplied on each fragment. The notation for chromophores, site states, and ESDs is the same as in (a). The Roman numbers denote the excitation rank between the two involved ESDs, and thus which ECI equation is used to calculate the matrix element. Numbers 0, I, and II correspond to eqs 8–10 [each with modifications (18)], respectively. The blocks **H**_**GS–GS**_, **H**_**LE–LE**_, and **H**_**DLE–DLE**_ are denoted by yellow, red, and blue frames, respectively.

When constructing the excitonic basis in ECI, we allow it to contain GS, LE, and MLE products, built from the site states with any *s* and *m*_*s*_ values, while MLE products can have any number of excited site sites. However, the size of this (full ECI) basis scales as
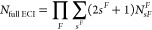
where  is the number of site states of spin *s*^*F*^ provided on site *F*. As can be seen, *N*_full ECI_ grows very quickly and can easily become too large to be handled. In analogy with regular CI, the GS product in ECI plays the role of the *aufbau* configuration, the LE products play the role of excitonic single excitations, and the MLE products play the role of excitonic double (DLE), triple (TLE), and higher excitations. Thus, like in CI, we can truncate the full ECI basis according to the excitation rank of the ESDs, obtaining the methods ECIS, ECISD, and so on. Full ECI is then ECISD ... *M*, while the basis used in the FEM corresponds to the ECIS expansion. The truncation of the ECI basis is schematically illustrated in Figure 2a. Here, we stress that the full ECI basis is still not complete, as it lacks the CT products, which would not have any analogy with the Slater determinants.

The full ECI Hamiltonian matrix has the block structure
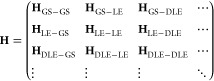
19where blocks like **H**_GS–TLE_, **H**_LE–QLE_, etc., are zero according to the excitonic analogue of the Slater–Condon rules. The detailed structure of an exemplary Hamiltonian matrix—as well as which ECI formulas are used to calculate which matrix elements—is illustrated in Figure 2b. In excitonic terms, the block **H**_GS–LE_ contains the GS–LE couplings, which are neglected in the FEM, but not a priori in ECI. Note that, unlike in the FEM, we also in principle consider the couplings between two LEs on the same site (those are part of **H**_LE–LE_). Both types of couplings are covered by the modified expression (9), although we note that this expression is more general and covers the couplings between any ESDs differing in one site state, regardless of their excitonic excitation ranks (GS, LE, DLE, etc.). Similarly, the modified expression (10) covers the couplings between LEs on the different sites (the ones also accounted for in the FEM) but additionally covers the couplings between any other pair of ESDs differing in exactly two site states.

As an example of computing coupling elements, consider  in Figure 2b. Here, the hexagon fragment changes electronic state, while the pentagon fragment stays. For this “differ-by-one” matrix element, we use eq 9 with modifications (18) and evaluate integrals by eqs 6 and 7. The coupling thus has three contributions: (i) the Coulomb interaction of the purple-to-green transition density of the hexagon fragment with the embedding-corrected nuclear charges of the pentagon fragment, (ii) the Coulomb interaction of the purple-to-green transition density (hexagon) with the white (ground state) density (pentagon), and (iii) the exchange interaction corresponding to (ii). The coupling element  also differs in one site state and hence is calculated with the same equations, where solely the white (ground state) density of the pentagon fragment is replaced by the purple (excited state) density. Both matrix elements use the same embedding charges of the pentagon fragment.

Instead of the matrix notation in eq 19, the ECI Hamiltonian can also be written similarly as
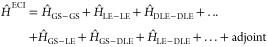
20All the constituent terms of the ECI Hamiltonian are given explicitly in Section S1 in the Supporting Information, where we also compare the ECI Hamiltonian to the FEM Hamiltonian in eq 11. As can be seen from Section S1, the Hamiltonian of the FEM (and of FEM-like models^16−19,27−29^) only contains a part of  and  and neglects all other terms. As mentioned in Section 2.2, some FEM-like models^18,19,35^ expand their Hamiltonian to include CT configurations, which are missing in  as discussed here. We also note that  (see Section S1) in some of the FEM-like models^27,28^ is corrected with respect to the nonorthogonality of the site molecular orbitals.

At this point, we want to briefly compare ECI to some of the “exact” fragment-based methods mentioned in the introduction. ECI in principle follows a philosophy similar to ref (20) but fully relying on the strong orthogonality assumption. This facilitates the construction of the full-system Hamiltonian only from site energies and 1-particle densities, making ECI simpler, cheaper, and more easily combinable with any site-state electronic structure method, as will be discussed below in Section 3.2.6. Another comparable method is the active space decomposition approach by Parker et al.^23,24^ This approach also requires only 1-particle site quantities. An important difference is that this approach employs a full-system SCF calculation followed by orbital localization to implicitly enforce strong orthogonality. In contrast, ECI never requires any kind of full-system calculation (or even dimer calculations), making it more affordable for systems with many fragments. Additionally, in the present work, we show that a smart choice of embedding charges (see Section 3.3) can make ECI converge rapidly with the number of site states. Therefore, and because ECI can be hierarchically truncated (ECIS, ECISD, etc.), we do not require a DMRG-based algorithm, but the full-system Hamiltonian can be rather diagonalized directly, as common for exciton models.

#### 3.2.2 Strong Orthogonality Violation

If the chromophores are spatially close, the assumption that all ESDs are strongly orthogonal is likely not fully warranted. However, even in this case, not all ESDs will violate the strong orthogonality assumption equally. Thus, prior to the construction of the matrix **H**, one should attempt to detect the ESDs that violate this assumption most significantly and expel them from the ECI basis. This scenario can easily occur if the site states on each chromophore are provided in a black-box manner (e.g., the first *N*_0_^*F*^ singlet states, the *N*_1_^*F*^ triplet states, etc.). The lobes of some of those site states might significantly penetrate into the regions of the other fragments, and thus the ESDs including those site states might violate the strong orthogonality assumption more significantly than the others. The most pronounced example of such site states would be Rydberg excited states. To track such site states, one can use the integral
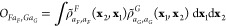
21as a measure how much site states  and  are “overlapping” (see eq 14.5.7 in ref (26)). Using this measure, we expel an ESD if it contains any pair *Fa*_*F*_, *Ga*_*G*_ of site states for which
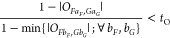
22

The idea behind this equation is to compare the overlap of a certain site-state pair with the smallest site-state overlap for the given pair of fragments and to request that the ratio of those two is not lower than a threshold *t*_O_. It is reasonable to keep this threshold slightly lower than 1. In the limiting case of *t*_O_ = 1, each site-state pair in an ESD would have to have the same overlap as the least-overlapping site-state pair for the respective pair of fragments, in order for the ESD to be accepted in the ECI basis. On the other hand, for *t*_O_ = 0, no truncation of the ECI basis due to the site-state overlapping would occur.

#### 3.2.3 Spin Adaptation of the ECI Basis

After the ECI basis is truncated due to strong orthogonality violations, it is convenient to perform spin adaptation considering the set of desired multiplicities. In particular, the ESDs are not necessarily eigenfunctions of the operator of the full-system electronic spin , and thus not all of them will contribute to the full-system states of a desired multiplicity 2*S* + 1. However, each ESD has a well-defined spin projection value . For a nonrelativistic full-system Hamiltonian, there is no coupling between two ESDs with different *M*_*S*_, i.e., the Hamiltonian matrix is block diagonal, with one block for each value of *M*_*S*_. For this reasons, when one calculates the full-system states of spin *S*, it is sufficient to diagonalize only a single block, corresponding to any *M*_*S*_ value in the range −*S*, −*S* + 1, ..., *S*. The most convenient choice is *M*_*S*_ = *S*, as this block contains the fewest ESDs for a given *S*. After such a block is diagonalized, only states of the chosen *M*_*S*_ are obtained—if the quantities related to the states of other *M*_*S*_ values are desired (e.g., spin–orbit couplings), they could be calculated by means of the Wigner–Eckart theorem.^36−38^ Taking only ESDs with *M*_*S*_ = *S* into the ECI basis trims the basis substantially and also reduces its scaling with the number of different multiplicities of the provided site states, decreasing computational cost.

Furthermore, not all ESDs with *M*_*S*_ = *S* will contribute to the full-system states of spin *S*, so we can further trim the ECI basis for spin *S*. We accomplish this by constructing the matrix of the  operator in the basis of the ESDs with *M*_*S*_ = *S* for each desired *S* separately. After diagonalizing, we obtain the excitonic configuration-state functions (ECSFs)—the eigenfunctions of . Then, we take only those ECSFs that correspond to the desired *S*, and all ESDs contributing to those ECSFs. For the details of the procedure, the notation used for ECSFs, and illustrative examples of ECSFs written in the basis of ESDs, see Appendix A. Finally, we calculate **H** in the spin-adapted ECI basis of ESDs, for each *S* separately. Before diagonalization, we transform the matrix into the basis of the ECSFs. In this way, we save some additional computational time because the number of ECSFs is always lower than or equal to the number of ESDs for a given *S*, and we also directly obtain the full-system states in the most compact form, easing analysis and interpretation. We note that, in principle, one could derive and use analytical formulas for the generation of ECSFs from ESDs, e.g., analogous to the genealogical coupling approach.^39^ However, the extension of such an approach to fragments with arbitrary spin would go beyond the scope of this work, so for the sake of simplicity, here we carry out the transformation by direct diagonalization of the  operator.

#### 3.2.4 Calculation and Characterization of the Full-System States

After the full-system Hamiltonian matrix (in the truncated and spin-adapted ECI basis) is calculated, the full-system states are obtained by diagonalization of that matrix (in practice separately for each spin block)
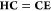
23Here, **C** is the matrix of ECI coefficients (coefficients of the full-system states written in the ECI basis), while the diagonal matrix **E** contains their energies. There is no overlap matrix between the ECSFs because the strong orthogonality assumption implies their (full) orthogonality. We also note that the construction and diagonalization of an effective Hamiltonian matrix is expected to produce proper adiabatic states with a formally correct topology of conical intersections and avoided crossings (as long as the underlying site-state method has this property). This property of ECI could be explored in more detail in future work.

Depending on how strongly the chromophores are interacting in the system, the ECI vectors can be more or less “correlated”. For example, if the individual ECSFs are already good approximations of the full-system states, the ECI coefficient of the leading ECSF will be very dominant (e.g., ≈±0.99) for each state. For systems with symmetry-equivalent fragments, one will usually find several symmetry-equivalent ECSFs with (up to sign) equal contributions, whose sum of squares is approximately 1. Such states dominated by one ECSF (or set of symmetry-equivalent ECSFs) are expected to occur in systems with weakly coupled fragments. On the other hand, if the individual ECSFs are not good approximations of the full-system states, each ECI vector will have a large number of non-negligible coefficients, i.e., a substantial “excitonic correlation” can be expected. In this case, one could speak of a strongly coupled multichromophoric systems.

However, whether one encounters weak or strong excitonic correlation does not only depend on how strongly the fragments are interacting but also depend on how the site states were constructed. In particular, excitonic correlation in ECI depends on the specific choice of the embedding charges, just like electronic correlation in (uni)molecular systems depends on the specific set of MOs used in the CI calculation. It should be stressed that, in our approach, excitonic correlation is also of relevance for the ECI ground state, which might exhibit some contributions of LE and MLE products (through GS–LE and GS–MLE couplings) in addition to the (likely dominating) GS product. Such excitonic correlation of the ground state is not possible in FEM and FEM-like models, as they neglect GS–LE couplings—in these models; the ground-state wave function is always equal to the GS product.

To track the amount of excitonic correlation in each full-system state, we define the participation ratio (PR) for full-system ECI state *I*
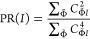
24where the sums go over the entire ECI vector of state *I* in the ECSF basis. This diagnostic will be equal to 1 if only a single ECSF contributes to the state (with ). If *L* ECSFs contribute with equal absolute values of ECI coefficients, PR will be equal to *L*, whereas if the ECI coefficients of the ECSFs are not equal, PR will have a value between 1 and *L*. Hence, it provides an estimation of the number of contributing ECSFs to the ECI vector. Further, to track the characters of the states in the terms of different excitation ranks (GS, LE, DLE, ...), we define the E_*n*_ diagnostics for excitation rank *n* as
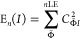
where *n* = 0 corresponds to the aufbau ECSF, *n* = 1 to all LE ECSFs, *n* = 2 to all DLE ECSFs, etc. Consequently, E_0_(*I*), E_1_(*I*), ... give the contribution of the corresponding ECSFs to the full-system state *I*.

#### 3.2.5 Calculation of the Full-System Properties

After obtaining the ECI coefficients, one can in principle easily calculate any unrelaxed property of the full-system states corresponding to a one-electron operator by means of the expectation value. In this work, we restrict ourselves to only the (transition) dipole moments (between) of full-system states, which are calculated as
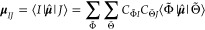
25where *I* and *J* are two full-system states, Φ̃ and Θ̃ are two ESDs, and  and  are the corresponding ECI coefficients (in the ESD basis). With Φ̃ given in eq 17, the (transition) dipole moment between two ESDs within the strong orthogonality assumption is only nonzero for  or for 
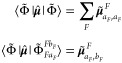
26where  is the dipole moment of the embedded site state , while  is the transition dipole moment between  and . Therefore, to obtain the electronic spectrum of the full system, one has to provide the embedded site energies, the site state/transition density matrices (which are in practice given by site-specific geometry, atomic basis set, and density matrix coefficients, see Appendix B), and all (transition) dipole moments of the site states, for each chromophore.

#### 3.2.6 Flexibility of the Site States

The ECI formulas are obtained with the assumption that the embedded site states are the exact eigenstates of the embedded site Hamiltonians (see eq 16). However, they actually also hold if an approximate electronic structure method is used in the site-state calculations, as long as it is variational. If the method is not variational, the embedded site energies  in eq 8 should formally be replaced by , while eq 9 should additionally contain  This can be seen in the first two equations in expression 14.1.7 in ref (26). These matrix elements can be evaluated from the site-specific 1- and 2-particle (transition) density matrices. However, if the employed method is accurate (i.e., if its states are sufficiently close to the exact eigenstates), one can expect  ≈  and  ≈ 0. This approximation would be very convenient, as it would circumvent handling large 2-particle density matrices. With this approximation for nonvariational methods, the ECI formulas could be used with any electronic structure method for the site-state generation.

As a matter of fact, employing the replacements given in the previous paragraph, the ECI formulas hold generally. In other words, any set of (proper quantum mechanical) functions of *N*_el_^*F*^ variables can be used as the set of site states, i.e., the site states do not even have to be approximations of the eigenstates of the site Hamiltonians. The only requirement is that the site states themselves are already spin-adapted, i.e., that they are eigenstates of the site-specific spin. This property allows one to, e.g., employ site-specific diabatic states as the site states in the ECI, if those are available and one wishes to avoid the (presumably expensive) calculation of the adiabatic site states. However, for diabatic states, the site-specific diabatic couplings  would be far from zero and would need to be included.

Given that ECI is constructed to be (approximately) agnostic to the underlying site-state electronic structure method, we expect that one could in principle also employ different methods for different fragments in the same ECI calculation. Such an approach would enable efficient mixed-accuracy multiscale calculations of extended systems. Here, the most challenging fragments could be treated with accurate methods that, e.g., properly describe doubly excited site states or multireference situations, whereas ancillary fragments could be treated with much cheaper methods.

Fragments treated with time-dependent density functional theory could also be included in the same way. However, this would bring two additional approximations with respect to the corresponding direct calculation—the interfragment exchange would be described by the ECI *K* terms (corresponding to the “Hartree–Fock” functional), while the interfragment correlation would be accounted for by the diagonalization of the ECI Hamiltonian. In contrast, in the direct calculation, both of those would be described by the employed exchange–correlation functional, i.e., on the same footing as exchange and correlation within the site.

### 3.3 Excitonic Hartree–Fock

The ECI formulas work for any (arbitrary) choice of embedding charges. Therefore, the embedding charges can be viewed as tuning parameters that only modify the site states (and hence the ECI basis) without modifying the full-system Hamiltonian operator (as it does not depend on the embedding charges, see eq 13). In particular, we stress that the embedding charges in ECI are not used to compute the interfragment interaction energy within the diagonal matrix elements (unlike in the above-mentioned exciton models^16,17^), as the ECI formulas already contain terms taking into account all contributions to matrix **H**. Nevertheless, due to their influence on the site states, embedding charges are expected to still strongly influence the overall performance of ECI. This is analogous to the effect of the choice of the MOs to the performance of a CI calculation.^40,41^

Consequently, the question arises what is the best choice of embedding charges. One possible choice, following the logic of the FEM, is to set the embedding charges of all atoms in the system to zero, which results in ECI with FDA. In this case, the ECI formulas (applying eq 18) simplify to the original GFT formulas (eqs 8–10). Another choice is to use partial atomic charges that reproduce the electrostatic potential of the individual chromophores in their ground states (the so-called ESP charges).^42,43^ These can be obtained by various regression procedures applied on the ground-state density,^42−46^ where the most stable one is restrained electrostatic potential (RESP) fit.^47^ This choice would result in ECI with similar embedding employed in the described exciton models employing the QM-MM electrostatic-embedding scheme^16,17^ since the force-field point charges are usually constructed as the ESP charges of the molecular ground state.^48^

However, in an analogy with McWeeny’s variational calculation for a single antisymmetrized product (described at the end of the Section 2.1), a better choice should be the set of embedding charges that minimize the full-system ground-state energy (*E*_0_), using the embedded GS product
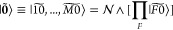
27as the test function. The variational energy of the product is given by eq 8 with modifications (18). Note that if the GS product is not an eigenstate of , it should be spin-adapted before being used as the test function. In this case, the spin-adapted GS product is a linear combination of several ESDs, and hence, its variational energy will be a linear combination of matrix elements that can be calculated using the ECI formulas.

In any case, apart from the condition that the embedding charges minimize *E*_0_, we also desire that they mimic the perturbation that one fragment causes to other fragments in the full system. Hence, we force the embedding charge of each atom to be equal to the embedded ground-state ESP charge of the atom. Under this restriction, the variational optimization of *E*_0_ can be solved by the following iterative procedure.

1. Select an initial guess for the embedding charges of all atoms in the system.
2. Calculate the embedded site ground state  for each fragment *F*.
3. Generate all embedded ESP charges *Q*_*f*_^*F*^ by RESP fit of  for each fragment *F*.
4. Set the new embedding charges to the calculated ESP charges for each atom in the system.
5. If not,  for each atom in the system, where *t*_*Q*_ is a prechosen threshold, return to step 2.
6. Using the final *Q*_*f*_^*F*^ s, evaluate the ground-state energy by eq 8 with modifications (18).

This procedure is equivalent to McWeeny’s self-consistent procedure, with the simplification that the ground state of one fragment is not directly affected by the exact ground-state mean field of the other fragments, but rather by the field of the ESP atomic charges of the other fragments’ ground states. It is also analogous to the so-called “freeze-and-thaw” procedures in frozen density embedding,^49−51^ but again in our approach, the electrostatic interaction is described via the ESP charges.

After the self-consistent ESP charges are obtained, they can be used in the generation of all (ground and excited) embedded site states for the ECI calculation, and hence, all site states will be affected by the same embedding. This idea of using the self-consistent ESP-based embedding has already been employed in the exciton model of Morrison et al.,^28^ while here we only put it the in broader context, formally related to the framework of the GFT. Namely, embedded site states calculated with the self-consistent ESP charges are the excitonic analogues of the canonical HF orbitals, given that these site states are obtained by considering the mean-field interaction between fragments in their *aufbau* configurations in a self-consistent manner. For this reason, we call the presented procedure excitonic Hartree–Fock (EHF) and the self-consistent ESP charges EHF charges. Within the EHF procedure, we expect that a reasonable initial guess for the embedding charges is to set them all to zero, which implies that the initial embedded ground states will be equal to the ground states of the isolated fragments (as discussed for the FDA above). Then, at the end of the first EHF cycle, one obtains the ESP charges of the isolated fragments. After convergence of EHF, the embedded ground state of one fragment is then self-consistent with the ground states of all other fragments.

It is worth mentioning that the EHF method can be used not only to obtain favorable embedding charges for ECI but also as a stand-alone method for the treatment of the full-system ground state alone, by calculating the EHF energy as the expected value of the energy of the spin-adapted GS product.

As a side remark, we note that, if converged EHF charges are used as the embedding charges in an ECI calculation, the block **H**_GS–LE_ = **H**_LE–GS_^†^ is expected to be small due to the excitonic analogue of Brillouin’s theorem (see Chapter 14.3 in ref (26)). If some other choice of embedding charges is made, the subsequent ECI calculation is analogous to CI with some general (noncanonical) orbitals, so Brillouin’s theorem does not hold and the **H**_GS–LE_ block does not necessarily contain small values. Hence, ECI with non-EHF embedding charges not only employs a presumably less favorable variational basis but it is also expected to be less size extensive, which can affect its performance.

## 4 Computational Methods

### 4.1 Implementation

The presented EHF and ECI methods were implemented in Python within a local development version of the SHARC package.^52,53^ The minimal working code needed to reproduce the results presented in this work is given in a zip file in the Supporting Information. The complete and documented code will become publicly available in a future release of SHARC. The newly implemented SHARC–ECI interface is written in a way that other quantum chemistry interfaces of SHARC can directly be called to perform the site-state calculations and provide all necessary site-related data (site energies, density matrices, and dipole moments). The SHARC–ECI interface also performs the EHF calculations, where the required RESP fits are performed by the site-specific interfaces using our recent multipole RESP implementation.^54^

The retrieved site-specific data is collected by the SHARC–ECI interface and passed to the ECI class, which constructs the ECI basis, performs truncation and spin adaptation, calculates the effective ECI Hamiltonian, diagonalizes it, and finally calculates the full-system dipole moment matrix. Details about how the ECI formulas are practically evaluated are given in Appendix B. The computational efficiency of our implementation is discussed in Section 5.5.

To facilitate the communication of all site-specific data to the SHARC–ECI interface, also the employed SHARC quantum chemistry interface needs several modifications. For the application discussed below, we have modified the SHARC–Gaussian interface to provide the basis set information and density matrices from Gaussian and to construct the missing density matrices and transition dipole moments (see Appendix B for details). We note that the communication with the SHARC–ECI interface is kept general and modular, so that other quantum chemistry programs/methods can easily be used in the future. The modular interface framework will also enable mixed-accuracy calculations discussed in Section 3.2.6.

### 4.2 Application

ECI is intended as an approximation to a direct full-system calculation performed with an equivalent level of theory. Therefore, we assessed its performance by comparing the electronic spectra of **G**_**4**_ and **MgG_4_^2+^** obtained by ECI to spectra obtained from direct calculations of the full systems, rather than to the experimental data. We used the CIS/def2-SVP method in Gaussian 16^55^ due to its affordability and ease of interpretation. Also, because CIS is variational, it is not necessary to consider the additional modifications or approximations for nonvariational site states discussed in Section 3.2.6. We stress that in this case (using CIS as the level of theory in the site-state calculations), each LE ECSF is a linear combination of singly excited full-system configuration-state functions. Similarly, each DLE ECSF is a linear combination of doubly excited configuration-state functions having two excited orbitals belonging to the two different fragments. Hence, an ECISD calculation might in principle produce some qualitatively correct full-system states that are not accessible by a direct full-system CIS calculation. In fact, in some of the ECISD calculations on several geometries of the **G**_**4**_ and **MgG_4_^2+^** systems (presented below), we obtained several states with a dominant DLE character.

For each system, six variants of ECI were tested, obtained by combining ECIS or ECISD expansions with the three choices for embedding charges discussed in Section 3.3: zero embedding charges, embedding charges from RESP fits, and embedding charges from EHF. These three choices will be referred to as FDA, ESP embedding, and EHF embedding, respectively. In practice, they are achieved by conducting EHF calculations with zero cycles, one cycle, or until convergence, respectively. We note that in the FDA-based calculations of **MgG_4_^2+^**, we found that the Mg^2+^ charge perturbs the site states on guanines very strongly, such that setting the corresponding embedding charge to zero (as it should be done in FDA) leads to completely wrong results. Hence, in the computation of the site states for the FDA calculations, we included a +2 embedding charge on the Mg atom (all other embedding charges were zero).

Additionally, FEM calculations have been done for each system using the same level of theory and the same sets of site states as in ECI + FDA. As discussed above, ECIS + FDA is conceptually very similar to the FEM, with the only difference being that ECIS + FDA does not neglect the interfragment interaction energy on the diagonal, and GS–LE and the same-site LE–LE couplings. Nevertheless, as we will show below, the performance of ECIS + FDA and the FEM is substantially different.

Throughout the work, the threshold *t*_O_ from eq 22 was set to 0.95, while the threshold *t*_*Q*_ in EHF was set to 10^–4^*e*. In all ECI calculations, the set of site states on each guanine moiety comprised S_0_–S_7_ and T_1_–T_3_. For the Mg^2+^ fragment in **MgG_4_^2+^**, we included only the S_0_ site state because the excited site states occur at excitation energies higher than 50 eV and hence are not expected to influence the spectrum in the low-energy region in which the LEs of the guanines are found.

The employed geometries of **G**_**4**_ and **MgG_4_^2+^** were optimized in the ground state, using a full-system HF calculation, without the use of symmetry. The optimized geometries of **G**_**4**_ and **MgG_4_^2+^** were found to belong to the *C*_4h_ and S_4_ point groups, respectively, and were subsequently reoptimized in the largest subgroups available in Gaussian (*C*_2h_ and *C*_2_, respectively). Hence, all the four guanine molecules are symmetry equivalent in both systems, and therefore, they will all have the same site states. The excitation energies of the first eight singlet and first eight triplet excited states of the full system were compared, although we remind the reader that ECI and our implementation are formally capable of describing any spin multiplicity. For the excited singlet states, transition dipole moments and oscillator strengths from S_0_ were also calculated.

In order to examine the behavior of ECI and the FEM on nonsymmetric geometries, the electronic absorption spectra of **G**_**4**_ and **MgG_4_^2+^** were also calculated on five geometries sampled from the harmonic-oscillator vibrational Wigner distribution of the full-system ground state (at 0 K) computed with HF/def2-SVP. The optimized and Wigner-sampled structures are given in the Supporting Information zip file.

## 5 Results and Discussion

### 5.1 Site States

**Figure 3:**
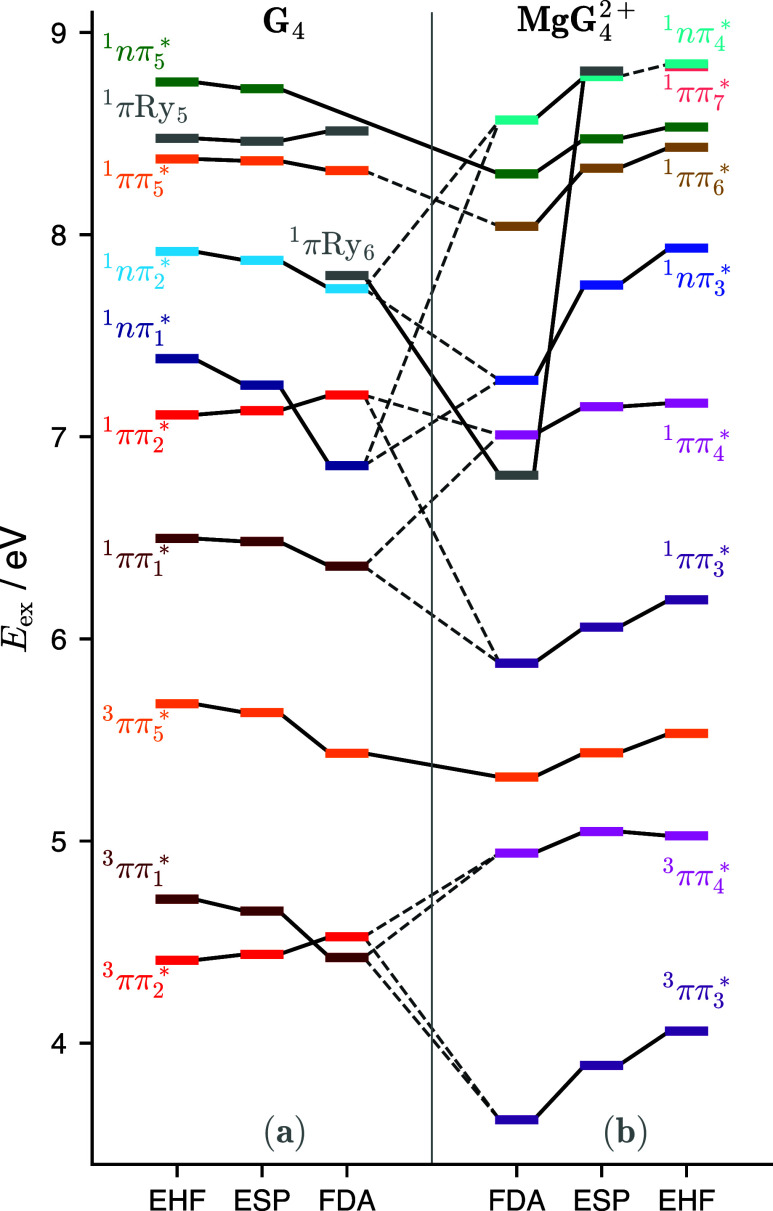
Comparison of excitation energies and electronic characters of the site states of guanine in (a) **G**_**4**_ and (b) **MgG_4_^2+^** systems with three different embedding schemes (FDA, ESP, and EHF). Full black lines match the states of the same character, while the dashed black lines match the states if they share only a portion of character.

The first step in an excitonic calculation is the computation of the site states. At the Franck–Condon geometries of **G**_**4**_ and **MgG_4_^2+^**, all guanine sites are symmetry equivalent, so that in this section, it is sufficient to discuss the site states of one guanine moiety. The vertical excitation energies and state characters are presented in Figure 3 for both systems and the three different embedding schemes (FDA, ESP, and EHF). Table S1 contains the corresponding excitation energies and oscillator strengths, while Table S2 contains the approximate (qualitative) expansions of the site states in the terms of molecular orbital excitations. Figure S1 displays all orbitals needed to define the characters of the site states.

In Figure 3, besides the excitation energies, we indicate the state characters and correlate states with the same character with lines. To compare the site states between the two different systems, the results using the FDA are located in the center; consequently, the figure has to be read “inside-out”.

In Figure 3a, with FDA, we find three triplet states at energies of about 4.5–5.5 eV. These states are of different ππ* characters (, ). Above 6 eV, we find seven singlet states that are of ππ* character—S_1_(), S_3_(), and S_6_()—of *n*π* character—S_2_() and S_4_()—or of Rydberg character—S_5_(^1^πRy_6_) and S_7_(^1^πRy_5_). When switching from FDA to ESP embedding, the excitation energies and state ordering change to some extent.  and , as well as  and , change their order. The swapped triplet site states are both of ππ* character, making it difficult to rationalize the influence of the embedding to their energy ordering. In contrast, the embedding-induced swap of the two singlet states can be traced to their different state characters. As can be seen in the figure, the  state is significantly shifted up in energy by the ESP embedding. This occurs because the  excitation shifts the electron density from the molecular plane to the perpendicular plane around the oxygen atom of guanine (see hole and particle orbitals in Figure S1). In **G**_**4**_, these oxygen atoms participate in hydrogen bonding with neighboring guanine in the molecular-plane configuration (see Figure 1). Thus, the  excitation weakens the hydrogen bonds, leading to an overall increase in the energy of .

In Figure 3a, at higher energies, we observe some more changes upon switching from the FDA to ESP embedding. While , , and ^1^πRy_5_ do not significantly change in energy, the ^1^πRy_6_ state character is strongly destabilized and is not found among the seven lowest singlets with ESP embedding. The latter state is destabilized because its particle orbital is located in front of the NH and NH_2_ groups that in **G**_**4**_ form hydrogen bonds with the oxygen atoms of the neighboring guanine. Hence, once the negative ESP charges of the neighboring oxygen are included, its repulsion with the Rydberg orbital leads to a strong destabilization of the ^1^πRy_6_ state. In turn, with ESP embedding, a new state character,  is observed as the S_7_ state.

**Figure 4:**
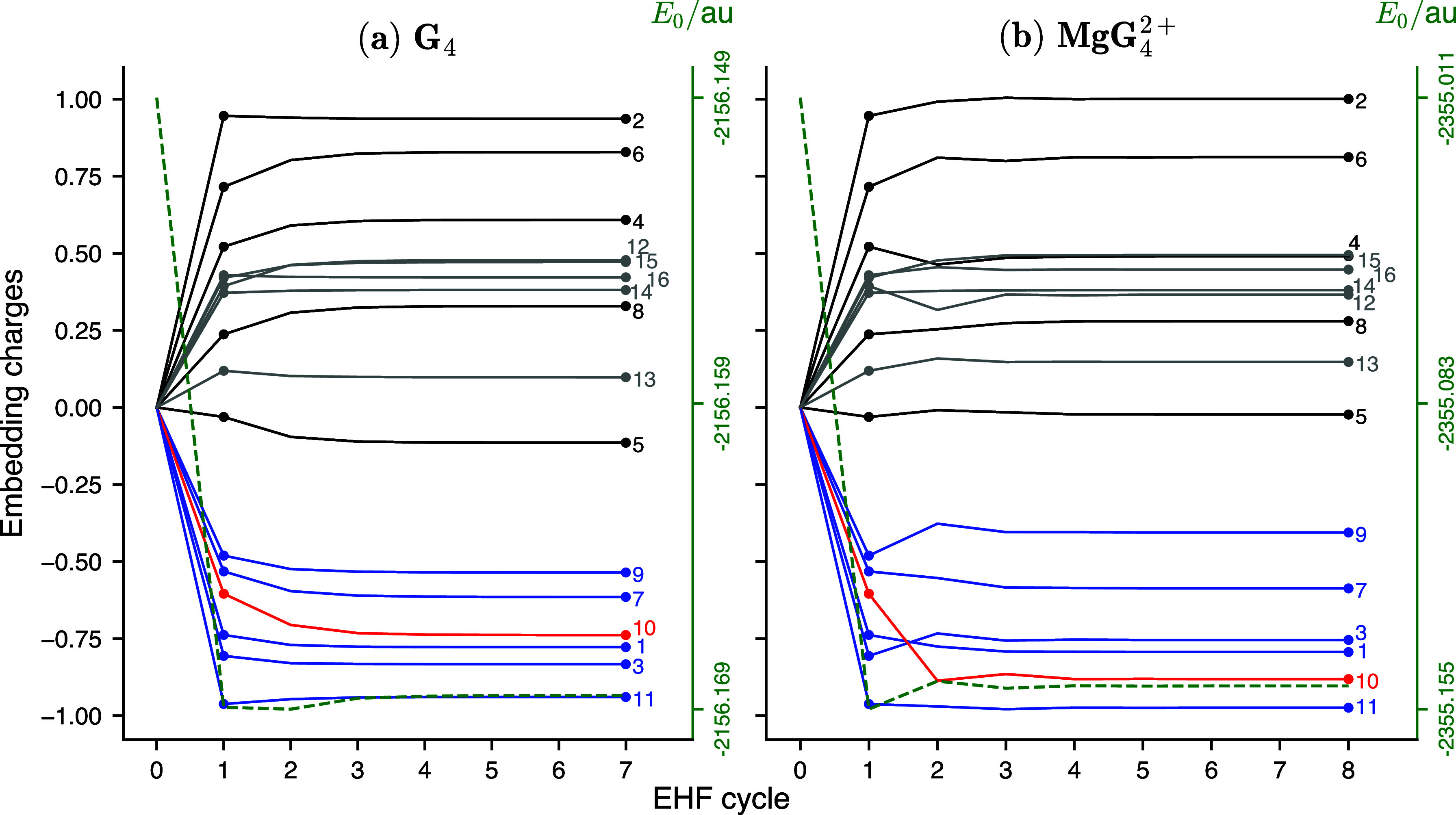
Convergence of the embedding charges of a guanine molecule in the EHF calculation on the symmetric Franck–Condon geometries of **G**_**4**_ (a) and **MgG_4_^2+^** (b) systems. Each line represents the ESP charge of a single atom of guanine (as guanine molecules are symmetrically equivalent, this is the same for each guanine unit). The atom numbers are given to the right of the corresponding lines (see atom numbering in Figure 1). Colors indicate element (black: C, gray: H, blue: N, and red: O). The dashed green line on each plot shows the convergence of the EHF energy (energy axis on the right).

Going from ESP embedding to EHF embedding in **G**_**4**_ only slightly modifies the excitation energies of the site states, leaving their energy ordering and characters untouched. The reason can be seen in Figure 4a, which shows that the embedding charges of the guanines in **G**_**4**_ do not significantly change between ESP and EHF (compare EHF cycle = 1 and EHF cycle = 7). The overall root-mean-square deviation (RMSD) of the ESP and EHF charges is only 0.069*e*. This, in turn, is because the fragments in **G**_**4**_ are relatively weakly interacting, and thus, the ESP charges of the isolated fragments are already rather close to self-consistency.

Figure 3b shows that FDA embedding with the +2 charge of Mg^2+^ in **MgG_4_^2+^** leads to mixing of the three pairs of site states with respect to the FDA embedding in **G**_**4**_. In particular,  and  mix to produce  and  (see Table S2 for the expansions of the site states). The same happens with the corresponding singlet states ( and  mix to form  and ). The third pair of mixing site states is  and  producing  and . It is also interesting to note that embedding with only the +2 charge of magnesium stabilized the ^1^πRy_6_ state, which now corresponds to the S_2_ state. This is due to the fact that the Rydberg density in front of the NH_2_ group is sufficiently close to the Mg cation to induce a stabilization effect via their attractive interaction.

This stabilization of the ^1^πRy_6_ state is so strong that it remains in the set of the computed seven singlet states even when ESP embedding is employed, which destabilizes the ^1^πRy_6_ state strongly due to the negative ESP charges of the neighboring oxygen atom. The other guanine site states in **MgG_4_^2+^** stay qualitatively the same when switching to ESP embedding, only changing their excitation energies to some extent. When EHF embedding is employed in **MgG_4_^2+^**, the ^1^πRy_6_ state is finally expelled from the first seven excited states and is replaced by new character, , which is found as the S_6_ state, while the S_7_ state is . These differences in the site states with ESP and EHF embedding in **MgG_4_^2+^** are due to the fact that in this system, the chromophores are much more strongly coupled, and therefore, ESP and EHF charges differ much more than in **G**_**4**_. The changes in embedding charges during the EHF cycles are shown in Figure 4b. Here, the difference between ESP and EHF charges (EHF cycles = 1 and 8, respectively) is particularly large for the oxygen atom, for which the ESP charge is −0.60*e*, but the EHF charge is −0.88*e*. Overall, the RMSD between the ESP and EHF charges is 0.084*e*.

### 5.2 Construction of the ECI Basis

Here, we briefly describe the construction of the ECI basis from the (embedded) site states for **G**_**4**_ and **MgG_4_^2+^**. Each guanine contributes a singlet ground site state, seven singlet excited site states, and three triplet site states. Each triplet site state actually contributes three *m*_*s*_ components, so that there are overall 16 excited site states per guanine. In **MgG_4_^2+^**, the Mg^2+^ fragment only contributes a singlet ground site state. For this reason, the **G**_**4**_ and **MgG_4_^2+^** systems have initial ECI bases (for each ECI expansion) of the same size, as the ESDs in the **MgG_4_^2+^** system are just antisymmetrized products of the ESDs of **G**_**4**_ and the ground site state of Mg^2+^. The number of ESDs that can be constructed from this set of site states and the corresponding numbers after applying the overlap criterion, spin adaptation, and multiplicity selection for both **G**_**4**_ and **MgG_4_^2+^** in all six ECI calculations are given in Table 1.

**Table 1:** Number of ESDs and ECSFs Encountered in the Construction of the Excitonic Basis for **G**_**4**_ or **MgG_4_^2+^** with Either Seven or Eight Singlet and Three Triplet States^a^

	7S + 3T		8S + 3T	
	ECIS	ECISD	ECIS	ECISD
*N*_0_	65	1601	65	1601
*N*_*O*__–__crit._	61	1411	65	1601
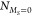	37	631	41	749
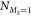	12	336	12	372
*N*_*S* = 0_	25/25	295/403	29/29	377/485
*N*_*S* = 1_	12/12	282/336	12/12	318/372

a *N*_0_: initial number of ESDs. *N*_*O*–crit._: number of ESDs after the overlap criterion is applied.  and : number of ESDs of the given *M*_*S*_ value. *N*_*S* = 0_ and *N*_*S* = 1_: number of ECSFs/contributing ESDs for a given spin *S*.

Given the set of site states, the ECIS basis (for both systems) initially contains 65 ESDs—one GS product and 16 (seven singlet and 3 × 3 triplet excitations) LE products per guanine fragment. In ECISD, additionally 16^2^ DLE products for each of the six pairs of guanines are included, for a total of 1601 ESDs. After applying the overlap criterion (eq 22) to the initial ECI basis, all ESDs containing the ^1^πRy_6_ state—regardless of system, embedding, or excitation rank—are expelled. This is expected because, as discussed, the lobes of the ^1^πRy_6_ state penetrate the region of the neighboring fragment, leading to a violation of the strong orthogonality assumption. The ^1^πRy_6_ state is only present in the FDA calculation of **G**_**4**_ and the FDA and ESP calculations of **MgG_4_^2+^**. Hence, these calculations were effectively performed with one less singlet state, leading to the numbers of ESDs and ECSFs labeled “7S + 3T” in Table 1. In particular, in the ECIS calculations without ^1^πRy_6_, only 61 ESDs are obtained (before considering spin), while in the ECISD calculation, 1411 ESDs were found. In the ESP and EHF calculations of **G**_**4**_ and the EHF calculation of **MgG_4_^2+^**, the problematic ^1^πRy_6_ state is not part of the site-state set, and no ESDs had to be expelled from the excitonic basis due to violations of the strong orthogonality assumption. Thus, these calculations were performed with the basis denoted as “8S + 3T” in Table 1 (65 ESDs for ECIS and 1601 for ECISD).

In the 7S + 3T calculations, out of the 61 remaining ESDs in ECIS, there are 37 with *M*_*S*_ = 0 and 12 each with *M*_*S*_ = ± 1. After spin adaptation, we obtain 25 singlet ESCFs (consisting of 25 ESDs with *M*_*S*_ = 0) and 12 triplet ECSFs (consisting of 12 ESDs with *M*_*S*_ = 1). In ECISD, out of the 1411 ESDs, 631 have *M*_*S*_ = 0, 336 × 2 have *M*_*S*_ = ± 1, and 54 × 2 have *M*_*S*_ = ± 2. Spin adaptation then produces 295 singlet ECSFs (consisting of 403 ESDs with *M*_*S*_ = 0) and 282 triplet ECSFs (consisting of 336 ESDs with *M*_*S*_ = 1), as well as 54 quintet ECSFs (consisting of 54 ESDs with *M*_*S*_ = 2) which are not used here. On the other hand, in the 8S + 3T calculations, slightly more ESDs and ECSFs were obtained, as given in the table. Note that in ECISD, the spin adaptation of ESDs having *M*_*S*_ = 0 does not only produce singlet ECSFs but also produce triplet and quintet ones, which could in principle be used to calculate triplet/quintet states with *M*_*S*_ = 0. Similarly, the spin adaptation of ESDs with *M*_*S*_ = 1 yields some quintet ECSFs. However, as discussed, it is sufficient to calculate states of only one *M*_*S*_ value for each multiplicity, and hence, these higher-spin ECSFs are discarded.

### 5.3 System **G**_**4**_

#### 5.3.1 Symmetric Geometry

Table 2 shows the comparison of excitation energies and oscillator strengths of the first eight triplet and eight singlet states of **G**_**4**_, calculated by the direct method, the FEM, and the six considered variants of ECI. The ECI vectors of the full-system states are given in Tables S3–S9. The direct (full-system CIS) calculation serves as reference. As shown in Table S10, the first 28 states in the direct full-system CIS calculation are all found to be guanine-centered excitations.

**Table 2:** Excitation Energies (and Oscillator Strengths from the Ground State for Singlets) of the First Eight Triplet and First Eight Singlet Excited States of **G**_**4**_, Calculated with the Direct Full-System Method, FEM, and Six Different Variants of ECI on the Frank–Condon Geometry^a^

state	direct	FEM	—FDA—		—ESP—		—EHF—	
			ECIS	ECISD	ECIS	ECISD	ECIS	ECISD
—Triplet Excitation Energies *E*_exc_ (eV)—								
T_1_	4.3949	4.4236	4.5078	4.4575	4.4191	4.4165	4.4083	4.4083
T_2_	4.3949	4.4236	4.5078	4.4575	4.4191	4.4165	4.4083	4.4083
T_3_	4.3949	4.4236	4.5078	4.4575	4.4191	4.4165	4.4083	4.4083
T_4_	4.3950	4.4236	4.5078	4.4575	4.4191	4.4165	4.4083	4.4083
T_5_	4.7381	4.5262	4.7315	4.6890	4.7061	4.7048	4.7138	4.7138
T_6_	4.7381	4.5262	4.7315	4.6890	4.7061	4.7048	4.7138	4.7138
T_7_	4.7381	4.5262	4.7315	4.6890	4.7061	4.7048	4.7138	4.7138
T_8_	4.7382	4.5262	4.7315	4.6890	4.7061	4.7048	4.7138	4.7138
RMSD		0.151	0.080	0.056	0.028	0.028	0.020	0.020
MAD		0.120	0.060	0.056	0.028	0.027	0.019	0.019
MSD		–0.092	0.053	0.007	–0.004	–0.006	–0.006	–0.006
—Singlet Excitation Energies *E*_exc_ (eV)—								
S_1_	6.4030	6.2740	6.5137	6.4244	6.4221	6.4184	6.4206	6.4206
S_2_	6.4883	6.3671	6.5213	6.4916	6.4894	6.4889	6.4909	6.4909
S_3_	6.4883	6.3671	6.5213	6.4916	6.4894	6.4889	6.4909	6.4909
S_4_	6.5262	6.4255	6.5691	6.5372	6.5346	6.5338	6.5354	6.5354
S_5_	7.0400	6.8571	7.1671	7.1139	7.0813	7.0774	7.0698	7.0698
S_6_	7.0686	6.8571	7.1954	7.1432	7.1109	7.1073	7.0998	7.0998
S_7_	7.0686	6.8571	7.1954	7.1432	7.1109	7.1073	7.0998	7.0998
S_8_	7.1530	6.8572	7.2766	7.2264	7.2001	7.1966	7.1897	7.1897
RMSD		0.183	0.100	0.053	0.032	0.029	0.024	0.024
MAD		0.172	0.091	0.042	0.025	0.023	0.020	0.020
MSD		–0.172	0.091	0.042	0.025	0.023	0.020	0.020
—Singlet Oscillator Strengths (from S_0_)—								
S_1_	0.0000	0.0000	0.0000	0.0000	0.0000	0.0000	0.0000	0.0000
S_2_	0.7436	0.7018	0.6287	0.6296	0.6412	0.6449	0.6445	0.6445
S_3_	0.7436	0.7018	0.6287	0.6296	0.6412	0.6449	0.6445	0.6445
S_4_	0.0000	0.0000	0.0000	0.0000	0.0000	0.0000	0.0000	0.0000
S_5_	0.0000	0.0000	0.0000	0.0000	0.0000	0.0000	0.0000	0.0000
S_6_	1.2532	0.0000	0.9644	0.9638	1.0811	1.0852	1.0961	1.0961
S_7_	1.2532	0.0000	0.9644	0.9638	1.0811	1.0852	1.0961	1.0961
S_8_	0.0000	0.0026	0.0000	0.0000	0.0000	0.0000	0.0000	0.0000
RMSD		0.627	0.156	0.156	0.100	0.097	0.093	0.093
MAD		0.324	0.101	0.101	0.067	0.067	0.064	0.064
MSD		–0.323	–0.101	–0.101	–0.067	–0.067	–0.064	–0.064

a Reported RMSDs, MADs, and mean signed deviations (MSDs) are with respect to the direct calculation.

According to the excitation energies, the FEM performs reasonably well on this system, yielding mean absolute deviations (MADs) of the excitation energies below 200 meV for both triplet and singlet states. However, the oscillator strengths of the states S_6_–S_8_ do not agree qualitatively with the reference—S_6_ and S_7_ should carry large oscillator strengths, but the FEM calculation yields zero oscillator strengths. Conversely, S_8_ should be forbidden but in the FEM acquires a small oscillator strength. These observations indicate that the FEM produces qualitatively wrong wave functions for those states.

Judging by the singlet excitation energies, ECIS + FDA performs slightly better than FEM, with MADs of the excitation energies about 60 meV for triplets and 90 meV for singlets. Interestingly, ECIS + FDA qualitatively reproduces the oscillator strengths of all computed singlet states with respect to the reference, whereas the FEM yielded wrong results for S_6_–S_8_. This difference can be explained by examining the characters of the FEM and ECIS + FDA states. According to the ECI vectors (Tables S3 and S4), FEM and ECIS + FDA agree on the description of the nearly degenerate S_1_–S_4_ system states of **G**_**4**_ as four linear combinations of the  LEs of the four guanines. In contrast, the S_5_–S_8_ states differ between the methods—the FEM predicts four superpositions of  LEs, whereas ECIS + FDA predicts superpositions of  LEs. This difference has a similar explanation as the changing order of the site states with different embedding charges in **G**_**4**_ (see Figure 3). In particular, the  excitation makes the O atom a worse hydrogen bond acceptor, so the excitation energy of  states should rise in the hydrogen-bonded full system. Indeed, ECIS + FDA captures this effect through the diagonal cross-fragment interaction terms in eq 8 (see also eqs S2 and S4). The FEM neglects those interaction terms and thus misses the change in hydrogen bond strength. The effect of neglecting the interaction terms in FEM can most easily be seen by comparing the site-state energies (Table S1)—4.423 eV for T_1_, 4.526 eV for T_2_, 6.359 eV for S_1_, and 6.857 eV for S_2_—with the FEM energies (Table 2). The close agreement (within few meV) shows that for **G**_**4**_, FEM predicts excitation energies dominated by the site-state energies.

Moving from ECIS + FDA to ECISD + FDA, the MADs of energies drop further for both triplets and singlets (to 60 and 40 meV, respectively). It appears that the excitonic doubles correct the descriptions of the dominantly LE states. The energetic ordering and degeneracy patterns are preserved, and the oscillator strengths are not affected by the addition of the excitonic doubles (compare ECIS + FDA and ECISD + FDA in Table 2).

Carrying out a single cycle of the EHF procedure, i.e., using ESP charges, yields a considerable improvement in excitation energies and oscillator strengths over the FDA. For ECIS + ESP, the MAD drops to 28 meV for triplets and 25 meV for singlets. This suggests that the quality of the site states—controlled by the choice of the embedding charges—has a larger impact on the performance of ECI than the extension of the excitonic basis by including excitonic doubles. Indeed, ECISD + ESP charges do not significantly improve the results over ECIS + ESP (MADs of 27 meV for triplets and 23 meV for singlets). In other words, ESP embedding produces site states of sufficient quality that an ECIS expansion can accurately describe the states of dominant LE character, and going to ECISD does not meaningfully improve the results (in contrast to the FDA calculations, where DLE products do matter).

The performance of ECIS can be further improved by employing EHF embedding. In this case, ECIS + EHF shows MADs of around 20 meV for both singlets and triplets. Thus, the EHF embedding prepared such site states that only GS and LE products are needed to construct the full-system states in almost perfect match with the direct method. The addition of DLE products by switching to ECISD + EHF yields negligible improvement, leaving the MADs untouched.

In general, in Table 2, we can observe that FEM tends to underestimate excitation energies, where ECI overestimates them. We assume that this is because FEM neglects all GS–LE couplings and thus underestimates the excitonic correlation of the full-system ground state. In ECI, in contrast, the GS–LE couplings stabilize the ground state, leading to larger excitation energies. We also observe in the table that all methods systematically underestimate the oscillator strengths. This might be explained because in all excitonic methods, we have computed only the unrelaxed transition dipole moments, according to eq 25.

#### 5.3.2 Nonsymmetric Geometries

**Figure 5:**
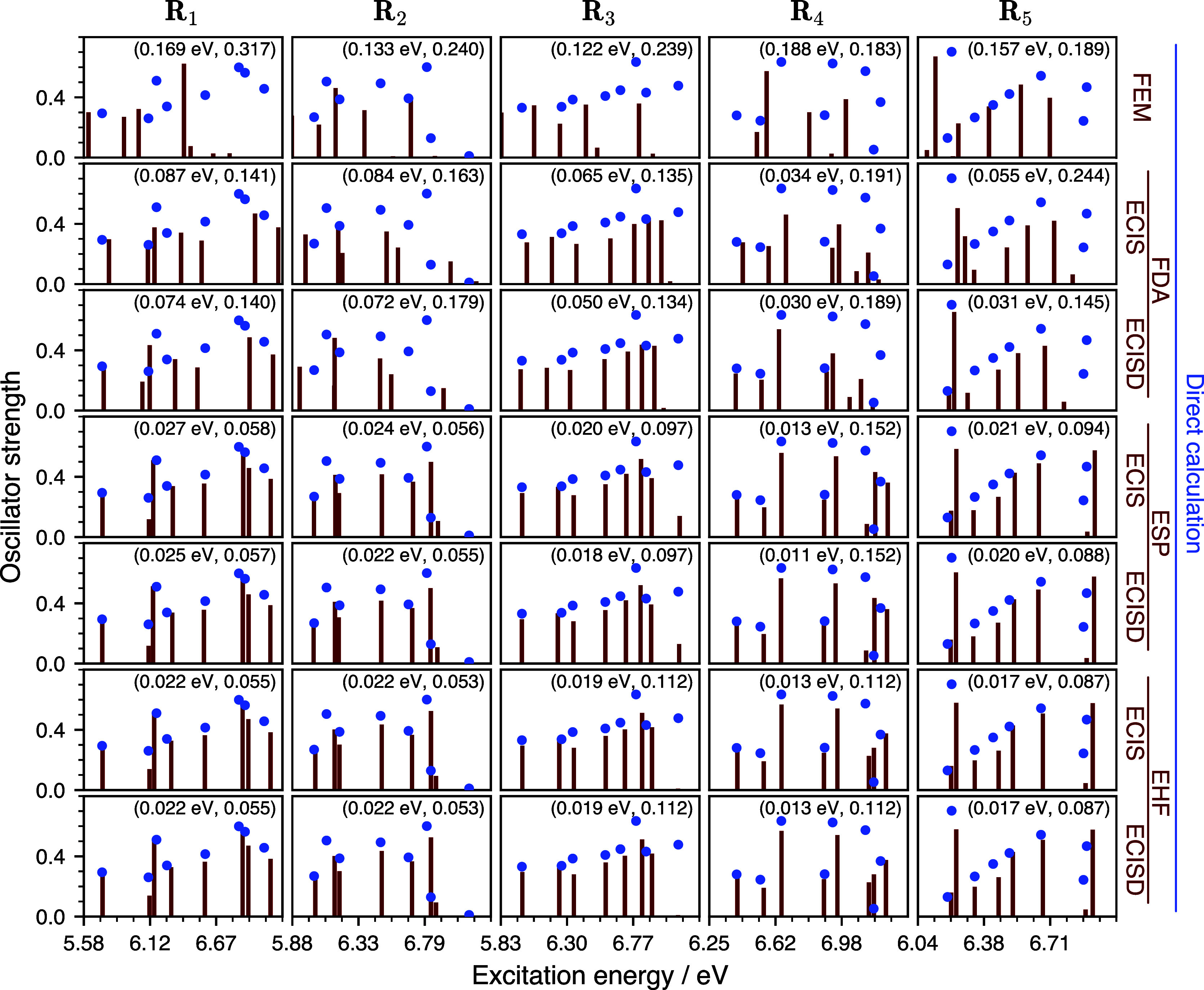
Comparison of electronic absorption spectra of **G**_**4**_ calculated with the direct method, the FEM, and the six considered variants of ECI (rows) on five geometries (**R**_1_–**R**_5_) sampled from the ground-state vibrational Wigner distribution (columns). Blue dots represent the direct-method results, while red lines represent the FEM and ECI spectra. The pairs of numbers at the top of each panel are MADs of the excitation energies and oscillator strengths.

Figure 5 shows the line spectra of **G**_**4**_ for five geometries sampled from the Wigner distribution to present the performance of ECI on nonsymmetric structures. All geometries belong to the *C*_1_ point group.

FEM and ECIS + FDA (first two rows) perform comparably well, having MADs of excitation energies between 0.1 and 0.2 eV. However, similar to the Frank–Condon geometry, the FEM spectra miss a lot of peaks and contain artificial peaks, while the ECIS + FDA spectra qualitatively reproduce most peaks of the direct calculation. Nevertheless, ECIS + FDA still exhibits significant numerical differences in energies, oscillator strengths, and state ordering with respect to the direct spectra. For example, in geometry **R**_1_, the relative ordering of states S_2_–S_4_ is apparently correct, but the oscillator strengths of S_2_ and S_4_ are too low. Furthermore, states S_6_–S_8_ have significantly too low oscillator strengths in ECIS + FDA, compared to the direct calculation, indicating qualitatively wrong state characters. A similar behavior of ECIS + FDA can be observed in the other four geometries. Arguably, the best agreement was reached for **R**_3_, where the state ordering and oscillator strengths are qualitatively correct, although ECIS + FDA shows a systematic blue shift and systematically lower oscillator strengths, compared to the reference. Here, we should note that the MADs are computed for the adiabatic states, so changes of state ordering of nearly degenerate states with very different oscillator strengths will lead to large deviations for the oscillator strengths, even though the obtained spectrum would be acceptable.

Going to ECISD + FDA brings similar improvements of the spectrum as in the symmetric geometry, cutting down the MAD values of both excitation energies and oscillator strengths for ≈30–40%. Interestingly, for geometry **R**_1_, both deviations increase. This observation will be discussed below, together with similar examples in the **MgG_4_^2+^** system (Section 5.4.2).

Employing ESP embedding systematically improves the performance of both ECIS and ECISD, cutting down the MADs of the excitation energies to <50 meV and of most oscillator strengths to below 0.1. This provides qualitative agreement with the direct calculations for all geometries, with the exception of states S_6_–S_8_ of **R**_5_. Again, just like on the Frank–Condon geometry of **G**_**4**_, when ESP embedding is used, there are only negligible differences between ECIS and ECISD because the ESP-embedded site states already provide a decent set of excitonic basis functions.

Notably, any remaining shortcomings of the ECI spectra are corrected when EHF embedding is employed. In this case, all states of ECIS and ECISD on all five geometries are both qualitatively and quantitatively correct, indicated by the MADs of only 20 meV for excitation energies and 0.07 for oscillator strengths.

### 5.4 System MgG_4_^2+^

While the system **G**_**4**_ has four identical chromophores, and the indexing of the fragments was irrelevant, the **MgG_4_^2+^** system additionally has an Mg^2+^ fragment. In the following, the four guanines in **MgG_4_^2+^** correspond to the indices *F* = 1, ..., 4, while Mg^2+^ is *F* = 5.

#### 5.4.1 Symmetric Geometry

Table 3 shows the comparison of the direct method, the FEM, and the six variants of ECI for the triplet and singlet states of **MgG_4_^2+^**. As said previously, in FEM and ECI + FDA calculations for this system, the embedding charge of Mg^2+^ was not set to zero but to +2. The corresponding energies and oscillator strengths for the “real” FDA (with embedding charge of Mg^2+^ also set to zero) calculations are given in Table S11. The ECI vectors of the full-system singlet states are given in Tables S12–S18. Table S19 compiles the results for 28 states computed with direct CIS. We find that the first eight states are guanine-centered states, whereas S_9_–S_12_ are guanine-to-metal CT states. Thus, using the first eight states for the comparison to ECI is appropriate.

**Table 3:** Excitation Energies (and Oscillator Strengths from the Ground State for Singlets) of the First Eight Singlet and First Eight Triplet Excited States of **MgG_4_^2+^**, Calculated with the Direct Full-System Method, FEM, and Six Different Variants of ECI on the Frank–Condon Geometry^a^

state	direct	FEM	—FDA—		—ESP—		—EHF—	
			ECIS	ECISD	ECIS	ECISD	ECIS	ECISD
—Triplet Excitation Energies (eV)—								
T_1_	4.0030	3.6208	4.7096	4.3186	4.1931	4.1183	4.0549	4.0586
T_2_	4.0031	3.6208	4.7096	4.3186	4.1931	4.1183	4.0549	4.0586
T_3_	4.0031	3.6208	4.7096	4.3186	4.1931	4.1183	4.0549	4.0586
T_4_	4.0033	3.6208	4.7096	4.3186	4.1931	4.1183	4.0549	4.0586
T_5_	5.0502	4.9401	5.4325	5.0893	5.0859	5.0296	5.0333	5.0359
T_6_	5.0505	4.9401	5.4326	5.0894	5.0859	5.0296	5.0334	5.0359
T_7_	5.0505	4.9401	5.4326	5.0894	5.0859	5.0296	5.0334	5.0359
T_8_	5.0508	4.9401	5.4326	5.0894	5.0860	5.0297	5.0334	5.0359
RMSD		0.281	0.568	0.225	0.137	0.083	0.039	0.041
MAD		0.246	0.544	0.177	0.113	0.068	0.034	0.035
MSD		–0.246	0.544	0.177	0.113	0.047	0.017	0.020
—Singlet Excitation Energies (eV)—								
S_1_	5.9465	5.7817	6.5980	6.2778	6.2032	6.1494	6.0943	6.0978
S_2_	6.0134	5.8431	6.6513	6.3210	6.2532	6.1950	6.1446	6.1480
S_3_	6.0134	5.8431	6.6513	6.3210	6.2532	6.1950	6.1446	6.1480
S_4_	6.2041	6.0327	6.8744	6.4400	6.4362	6.3314	6.3023	6.3053
S_5_	7.0940	6.9770	7.7288	7.3909	7.2745	7.2102	7.1542	7.1563
S_6_	7.1255	7.0076	7.7288	7.3909	7.2745	7.2135	7.1692	7.1711
S_7_	7.1255	7.0076	7.7653	7.4153	7.3019	7.2135	7.1692	7.1711
S_8_	7.1664	7.0399	7.9409	7.4230	7.3061	7.2460	7.2080	7.2094
RMSD		0.147	0.658	0.288	0.206	0.141	0.097	0.100
MAD		0.145	0.656	0.286	0.202	0.133	0.087	0.090
MSD		–0.145	0.656	0.286	0.202	0.133	0.087	0.090
—Singlet Oscillator Strengths (from S_0_)—								
S_1_	0.0103	0.0035	0.0003	0.0015	0.0000	0.0000	0.0020	0.0024
S_2_	0.6627	0.5271	0.4710	0.4605	0.4864	0.4862	0.5146	0.5153
S_3_	0.6627	0.5271	0.4710	0.4605	0.4864	0.4862	0.5146	0.5153
S_4_	0.0000	0.0000	0.0000	0.0000	0.0000	0.0000	0.0000	0.0000
S_5_	0.0000	0.0000	0.9395	0.8442	0.8343	0.0000	0.0000	0.0000
S_6_	0.9413	0.5981	0.9395	0.8442	0.8343	0.8208	0.9190	0.9193
S_7_	0.9413	0.5981	0.3057	0.0000	0.0000	0.8208	0.9190	0.9193
S_8_	0.2926	0.2065	0.0000	0.2724	0.2771	0.2703	0.3063	0.3023
RMSD		0.187	0.425	0.460	0.455	0.107	0.075	0.075
MAD		0.131	0.283	0.290	0.283	0.078	0.045	0.045
MSD		–0.131	–0.048	–0.079	–0.074	–0.078	–0.042	–0.042

a Reported RMSDs, MADs, and MSDs are with respect to the direct calculation.

The FEM calculation produces reasonable results, with MADs of the energy of 0.15–0.25 eV. However, it tends to underestimate all excitation energies (compared to the reference), in particular for the first four triplet states. It can be noted that in the direct calculation, the two sets of triplet states are marginally split (on the sub-meV order) in the expected 1:2:1 degeneracy pattern, whereas the FEM produces two sets of four degenerate triplet states. For the two sets of singlets, the degeneracy patterns and oscillator strengths are qualitatively correctly described by the FEM.

Although formally involving fewer approximations than the FEM, the ECIS + FDA method shows a significantly worse performance on **MgG_4_^2+^**. We find MADs of the energies of 0.5 eV for the triplets and 0.7 eV for the singlets, originating from a systematic overestimation of all excitation energies. The reason for this systematic blue shift is the inclusion of the GS–LE couplings, which are nonzero due to the FDA, because the site states are far away from self-consistency. In this system, these couplings are much larger than in **G**_**4**_ because Mg^2+^ strongly couples the GS product and any LE product through the interaction of the ground-state density (and the nucleus) of Mg^2+^ with the transition density on the excited guanine molecules. This coupling is described by a term in eq 9 corresponding to *G* = 5. The nonzero GS–LE couplings in **MgG_4_^2+^** lead to the loss of size extensivity, which in turn leads to an artificial increase in the splitting between the full-system ground state and the excited states, resulting in the aforementioned blue shift. In contrast, the FEM neglects the GS–LE couplings from the outset and therefore is in principle size extensive. Nevertheless, the S_1_–S_4_ states on the ECIS + FDA level seem to be qualitatively correct, with the S_1_ state having a small nonzero oscillator strength, the degenerate pair S_2_–S_3_ being very bright, and the S_4_ state being dark. On the other hand, according to the oscillator strengths, S_5_–S_8_ states are not qualitatively correct.

The ECI wave functions of those states (Table S13) nicely display the high excitonic correlation in this system when the FDA is employed. S_0_ exhibits about 6% admixture of LE products (E_1_ diagnostic in the table) due to the non-Brillouin GS–LE couplings. The states S_1_–S_3_ are superpositions of four S_1_ LEs and four S_3_ LEs. This is quantified by the participation ratio PR in Table S13, which shows that S_1_ is dominated by four equally contributing excitations and S_2_/S_3_ by two such excitations. In contrast, the S_4_ state has significant coefficients for the GS product and the S_1_ and S_3_ LEs and some contributions from higher states. Consequently, the PR for S_4_ is much higher (about 7). The second set of singlet states (S_5_–S_8_) exhibits a similar degree of mixing. This also suggests that, in addition to the inadequacy of the ECIS expansion and the FDA embedding, the used set of site states might not be sufficiently converged (i.e., that too few site states are supplied per fragment) for the description of the higher excited states of **MgG_4_^2+^** system. In general, the strong mixing between GS and LE products, as well as between two same-site LE products, is again a consequence of the strong interactions between each guanine and Mg^2+^, giving rise to large *J* terms in eq 9.

Employing the ECISD + FDA level in **MgG_4_^2+^** reduces the MADs of the energies by more than a factor of 2 compared to ECIS + FDA (from 0.5–0.7 to 0.2–0.3 eV). This is because including DLE products mitigate one source of nonsize extensivity of ECIS + FDA—the noncomplete ECI expansion. Furthermore, they account for residual excitonic correlation in the excited full-system states that is missed by ECIS + FDA. As can be seen in Table S14, all excited full-system states have contributions of DLE products of about 4–5% (E_2_ in the table). These mostly come from , , and  ESDs, corresponding to the DLEs on each pair of guanines (guanine indices *F* and *G* being any two different numbers between 1 and 4). We note that, although the energies were improved by adding DLE products, the oscillator strengths did not improve significantly because the DLE products do not contribute to the transition dipole moments from the state dominated by the GS product.

Switching to ESP embedding, we see—just like in **G**_**4**_—a large improvement in the results for **MgG_4_^2+^**. ECIS + ESP produces MADs of the energies of 0.20 eV for singlets and 0.11 eV for triplets (overestimating the excitation energies much less than FDA). While the triplet states show the expected degeneracy pattern (compared to the reference), for the S_5_–S_8_ states, ECIS + ESP predicts a 2:1:1 pattern instead of a 1:2:1 pattern, and the splitting of these states is notably too small. Similarly, the order of the bright and dark states differs between the reference and ECIS + ESP. This is most likely caused by the residual GS–LE couplings, which affect only the energy of the totally symmetric linear combination of the LE excitations (and the ground state). Hence, whereas the totally symmetric excitation is S_5_ for the reference, it becomes S_7_ for ECIS + ESP (see Table S15). Nevertheless, we note that the GS–LE couplings are dramatically reduced in ECIS + ESP compared to ECIS + FDA. The ECIS + ESP full-system ground state only exhibits an admixture of 1% of LE products (compared to 5% for ECIS + FDA), and the S_7_ state contains only 0.3% admixture of the GS product. The reduction of GS–LE couplings is expected to improve size extensivity for ECI + ESP compared to ECI + FDA. Similarly, excitonic correlation in ECIS + ESP is strongly reduced, best visible in the PR values in Table S15. This disappearance of excitonic correlation when applying ESP embedding is analogous to the disappearance of multireference character of electronic states when appropriate rotation of molecular orbitals is applied.^56,57^ Nevertheless, the site states obtained by ESP embedding are still not adequate to form a sufficiently accurate basis for ECIS.

Hence, unlike in **G**_**4**_, going to ECISD + ESP in **MgG_4_^2+^** leads to additional improvements. The MADs of the energies further drop by 30–40%, to 0.07 eV for triplets and 0.13 eV for singlets. As visible from the degeneracy pattern and oscillator strengths, unlike ECIS + ESP, ECISD + ESP describes the ordering of the S_5_–S_8_ states correctly, as the dark, totally symmetric state (Table S16) is lowered in energy (becoming the S_5_), and S_8_ showing the correct oscillator strength (compared to the reference).

As can be seen in Table 3, switching to EHF embedding in this system significantly improves the performance of ECI, leading to MADs of only 0.03 eV (triplets) and 0.09 eV (singlets). This is in contrast to **G**_**4**_, where ESP embedding was already sufficient and EHF embedding did not significantly improve the results. Particularly, at the ECIS + EHF level of theory, all eight excited singlet states are qualitatively correct in terms of degeneracy pattern, energy splitting, and oscillator strengths. The oscillator strengths of states S_5_–S_8_ agree very well with the reference, although the ones for S_1_–S_4_ are still too low. As can be seen from the ECI vectors in Table S17, expectedly EHF embedding completely eliminates any excitonic correlation in the ground state, as the E_0_ diagnostic is 1.000. This reduction in excitonic correlation is due to the self-consistency of the site states, which translates into effectively vanishing elements in the GS–LE couplings according to Brillouin’s theorem. This shows that ECI with EHF embedding can be considered to be size extensive, whereas other embedding schemes might have size extensivity issues. However, we note that one of the advantages of ECI is that one can recover size extensivity in the limit of complete sets of site states and a full ECI expansion, regardless of the embedding scheme.

For **MgG_4_^2+^**, going from ECIS + EHF to ECISD + EHF does not notably change the results, as the site states are of such quality that the excitonic singles are sufficient to correctly describe the dominantly LE full-system states. Although ECI + EHF produces decent results in **MgG_4_^2+^**, we note that the obtained MADs are still larger than in **G**_**4**_. Given that the set of site states with EHF embedding and ECI expansion is converged, this residual error in the spectrum can only be prescribed to a certain degree of violation of the strong orthogonality assumption, which in **MgG_4_^2+^** system comes from the proximity of the Mg^2+^ cation and the guanines. In addition to that, the lack of CT products also to some extent contributes to this error, as they can contribute to dominantly LE states through LE–CT couplings.

#### 5.4.2 Nonsymmetric Geometries

**Figure 6:**
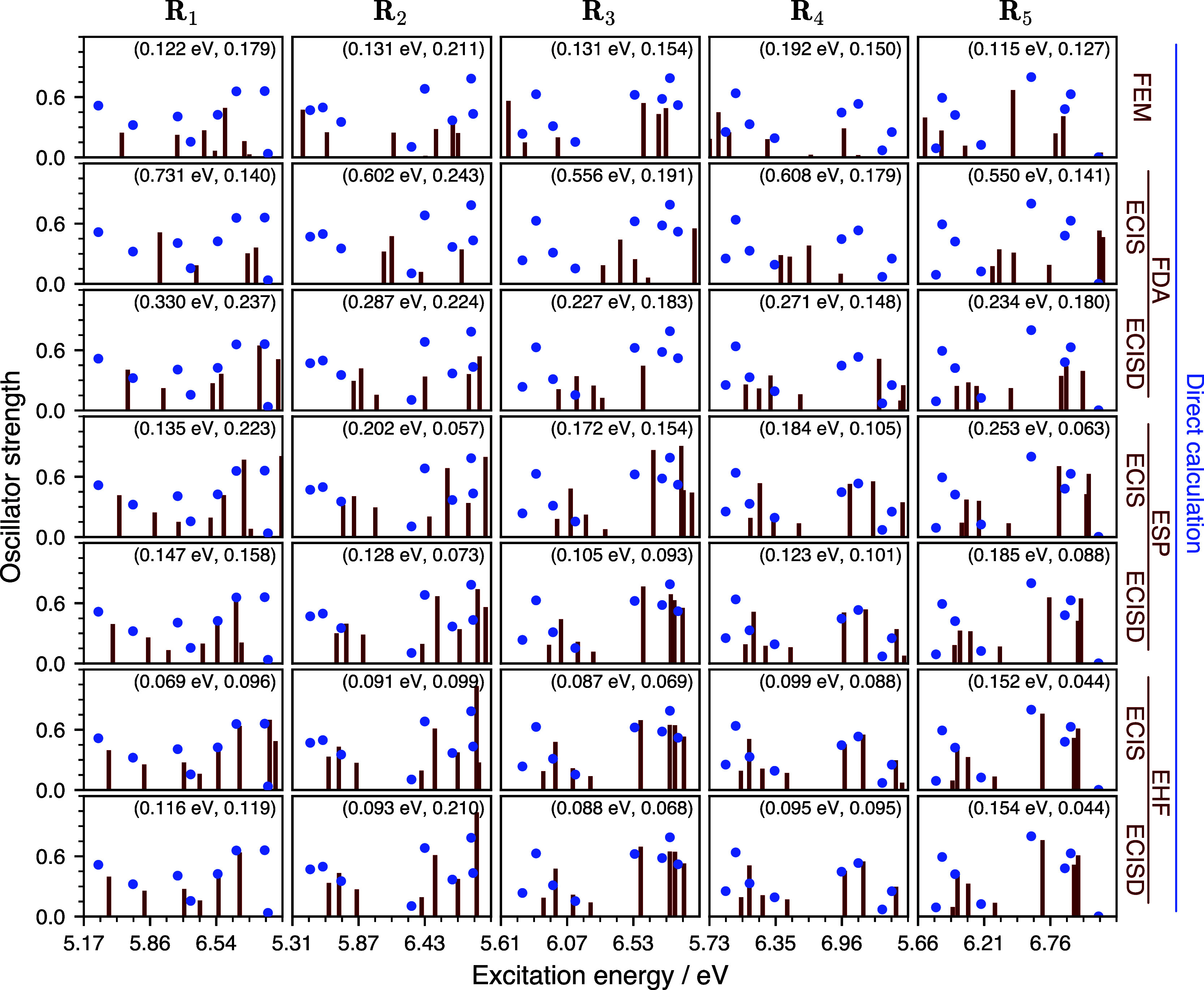
Comparison of electronic absorption spectra of **MgG_4_^2+^** calculated with the direct method, the FEM, and the six considered variants of ECI (rows) on five geometries (**R**_1_–**R**_5_) sampled from the ground-state vibrational Wigner distribution (columns). Blue dots represent the direct-method results, while red lines represent the FEM and ECI spectra. The pairs of numbers at the top of each panel are MADs of excitation energies and oscillator strengths.

Figure 6 shows the comparison of the electronic spectra of **MgG_4_^2+^** on five nonsymmetric geometries (analogous to Figure 5). Across the five geometries, the FEM and ECI methods behave generally similar to what was found for the Frank–Condon geometry. The FEM describes the low-lying states reasonably well but completely misses the high-lying ones. The ECIS + FDA method produces MADs in the energies of 0.5–0.7 eV, leading to qualitatively wrong spectra, just like at the Frank–Condon geometry. ECISD + FDA brings the errors down to about 0.2–0.3 eV, although it is apparent that the FDA is not appropriate for **MgG_4_^2+^**. ESP embedding further improves the results, with MADs of 0.14–0.25 eV for ECIS + ESP and 0.11–0.19 eV for ECISD + ESP, but still with some inaccuracies in the state ordering. These deviations are to a large extent due to the residual GS–LE couplings, as discussed above. Finally, EHF embedding gives satisfactory correct spectra, having MADs of 0.07–0.15 eV and having all peaks appearing in correct energy order.

For the ECISD-EHF calculation (and to a lesser extent, the ECISD + ESP calculation), however, we observe a somewhat surprising result. Specifically, for **R**_1_, the MAD significantly increases from ECIS to ECISD (0.07–0.12eV for ECISD + EHF). Similarly, for **R**_2_, the MAD of the oscillator strengths increases strongly when going from ECIS to ECISD. Visual inspection in Figure 6 shows that this is because the two highest computed states in these calculations “disappear”. A similar effect was already observed on the **R**_1_ geometry of **G**_**4**_.

To explain the qualitative change in the computed states, we examined their leading ECSFs in the ECIS and ECISD calculations and compared them to the direct full-system CIS calculation. The results are given in Table 4. As can be seen, ECIS predicts the S_1_–S_4_ states to be local S_1_ excitations on the four guanines, while the S_5_–S_8_ states are S_2_ LEs. Note that the geometry is not symmetric, and therefore, there is a single leading ECSF in each state, unlike in the symmetric Franck–Condon geometry. Also note that the direct full-system CIS calculation predicts nearly the same order of states, except that S_8_ and S_9_ are swapped.

**Table 4:** Excitation Energies and Dominant ECSFs of the First 11 Excited Singlet States of **MgG_4_^2+^** on Geometry **R**_1_ Calculated with ECIS + EHF, ECISD + EHF, and Direct CIS^a^

	ECIS		ECISD		CIS	
state	*E*_ex_	Ψ	*E*_ex_	Ψ	*E*_ex_	Ψ
S_1_	5.4316	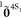	5.4342	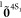	5.3235	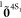
S_2_	5.7987	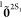	5.8011	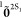	5.6817	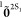
S_3_	6.2094	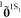	6.2126	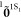	6.1443	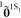
S_4_	6.3719	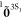	6.3749	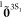	6.2789	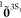
S_5_	6.5653	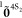	6.5019	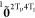	6.5589	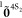
S_6_	6.7854	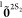	6.5671	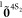	6.7516	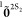
S_7_	7.0962	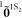	6.7865	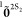	7.0448	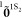
S_8_	7.1572	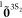	7.0415	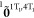	7.0786	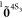
S_9_	7.1852	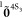	7.0985	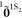	7.0991	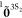
S_10_	7.5558	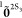	7.1050	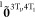	7.4420	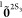
S_11_	7.6615	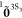	7.1588	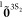	7.5391	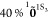
						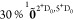
						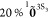

a The expansion of TT-DLE and CT ECSFs of overall singlet spin in the basis of ESDs is given in Appendix A. Dominant ECSFs were obtained from the leading ECI coefficients (all >0.95). For direct CIS, the corresponding wave function characters were obtained from visual inspection of the S_0_–S_*n*_ difference densities and with a CT number analysis in Theodore^58,59^ (Table S20).

In Table 4, when going to ECISD, we notice several additional states interleaved between the LEs. The S_1_–S_8_ states of ECIS are found as S_1_–S_4_, S_6_–S_7_, S_9_, and S_11_ in ECISD. The leading ECSF of the S_5_ state is , corresponding to a triplet–triplet double-LE (TT-DLE). This is a so-called singlet-fission precursor state,^60^ which is very dark (exactly dark within the strong orthogonality assumption) and hence does not appear in the spectrum. Likewise, the S_8_ and S_10_ states are also dominated by TT-DLE ECSFs. TT-DLE states are dominated by double excitations not only in the excitonic picture (dominated by DLE products) but also in the electronic picture (dominated by doubly excited Slater determinants). Hence, the TT-DLE states are not only inaccessible in ECIS but also in the direct CIS reference calculation. Validating their accuracy thus requires a correlated excited-state electronic structure method that properly includes double excitations, e.g., EOM-CCSD, ADC(3), or multireference methods. For a system like **MgG_4_^2+^**, such calculations are very challenging and go beyond the scope of this work. Nevertheless, we remark that ECISD is principally able to produce states of both LE and TT-DLE character on a formally equal footing.

### 5.5 Computational Cost

In order to assess the computational cost of ECI relative to the direct method, we conducted several additional calculations. To allow for a fair comparison, the number of states in the direct calculation and the number of site states for the ECI calculations were adjusted. Here, we observe that for both systems, the first eight full-system states S_1_–S_8_ are spanned almost exclusively by the first two LEs of each guanine. Hence, for assessing the computational cost, we included only the S_1_ and S_2_ site states of each guanine and computed the first eight singlet excited states, using ECIS, ECISD, ECISDT, and ECISDTQ. All four ECI expansions were combined with multiple EHF calculations with different convergence threshold *t*_*Q*_ from 10^–4^ to 10° (the last one is equivalent to ESP embedding). For comparison, for each system, we performed a direct full-system CIS calculation with Gaussian to compute eight states. All these calculations were run on one core of an Intel Xeon E5-2650 v3 CPU.

**Figure 7:**
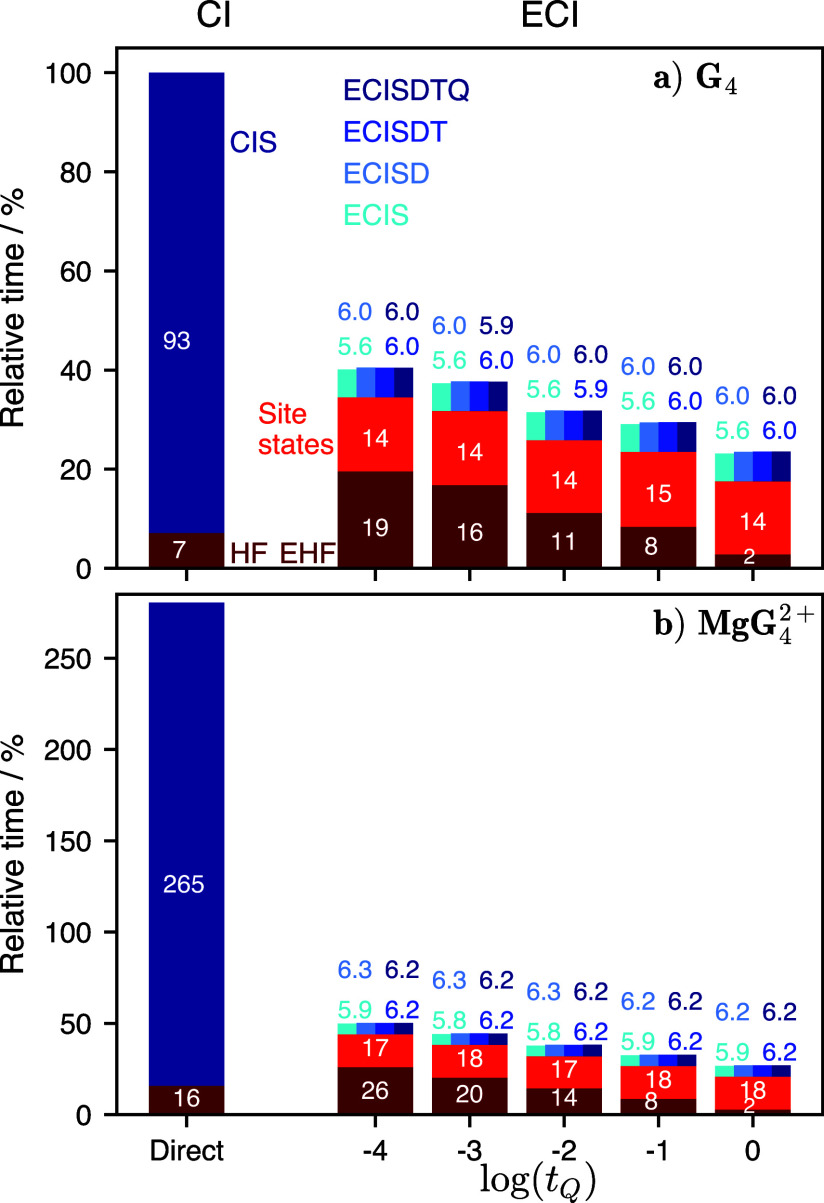
Relative time to compute eight excited states in direct full-system CIS calculations (left) as well as ECI + EHF calculations (right) for (a) **G**_**4**_ and (b) **MgG_4_^2+^**. The bars are split into their main contributions—for the direct calculations: HF (red, bottom) and CIS (blue, top), for the ECI calculations: EHF (red, bottom), site-state calculations (orange, middle), and ECI calculations (shades of blue for ECIS, ECISD, ECISDT, and ECISDTQ, top). The ECI + EHF calculations are conducted for different thresholds *t*_*Q*_ for the EHF convergence (*x* axis). Each calculation has been conducted on a single CPU core on the same machine. All timings are normalized to the cost of the direct full-system calculation of **G**_**4**_.

The obtained relative times are shown in Figure 7, split into the main calculation steps. For the direct full-system calculations, the timings are split into the HF and the CIS part. For the ECI calculations, they are split into EHF, site-state calculations, and the actual ECI part (construction of the ECI basis, spin-adaptation, evaluation of **H**, and diagonalization). Only the last part depends on the ECI expansion and therefore is given separately for the ECI expansions from ECIS to ECISDTQ. We note that the timings are similar for the different ECI expansions due to the small number of site states, but the results are still of similar quality as in Table 3 (see Table S21 for MADs).

Starting at the bottom, the converged EHF calculations are more expensive than the full-system HF calculation. However, the results in Table S21 indicate that already an EHF convergence threshold of 10^–1^ produces converged results, and the EHF calculation with that threshold has a similar cost as the full-system HF calculation. Furthermore, EHF scales better than full-system HF, and therefore, for the larger **MgG_4_^2+^** system, EHF is effectively not more expensive than HF.

The most expensive step in the full-system calculation is clearly the CIS part, taking on the order of few hours for the shown examples. We observe that the full-system calculation for **MgG_4_^2+^** takes approximately 2.8 times as long as the one for **G**_**4**_, demonstrating the rather steep scaling of the direct approach. The ECI calculations are significantly cheaper, taking 20–40% of the corresponding full-system calculation for **G**_**4**_ and 10–20% for **MgG_4_^2+^**. In Figure 7, only the EHF step depends on the EHF convergence threshold, whereas the site state and ECI steps do not. We observe that the overall ECI computation time changes by less than 20% from **G**_**4**_ to **MgG_4_^2+^** This is a clear sign of the more favorable scaling of ECI with respect to the full-system CIS calculation.

The test system that we use here is still rather modest, and we expect even larger speed-ups of ECI compared to full-system calculations for larger systems and for systems with more fragments. Moreover, ECI will become increasingly favorable the more unfavorable the scaling of the electronic structure method becomes. As an extreme example, if the guanine units were computed with CASSCF(14e,11o),^61^ an ECI calculation would be entirely feasible. On the other hand, a direct calculation would correspond to CASSCF(56e,44o), which is unfeasible. We note that in such a CASSCF-based ECI calculation, the cost of the ECI part would depend only on the number of basis functions and site states but not on the size of the active space. Only the EHF and site-state parts scale with the active space size.

There are several possibilities to further improve the performance of the EHF and ECI steps. First, as we have discussed above, ECI in principle works with any set of embedding charges, where more accurate embedding charges will produce better site states and thus will require less correlation treatment in the ECI step. Thus, the iterative EHF procedure could in principle be conducted using a different, cheaper level of theory than the site-state calculations. The only requirement would be that this cheaper level of theory provides a reasonable description of the ground-state charge distribution. For example, embedding charges from density functional theory might be used for CASSCF-based ECI. A more approximate but cheaper approach would be to employ constant, geometry-independent embedding charges, e.g., from a force field (as done previously^16,17^) or from a RESP fit at the equilibrium geometry. With this approach, the EHF part of the cost could be avoided.

Second, the ECI step can become the computational bottleneck for large number of site states, high spin multiplicities, and/or high excitation ranks. Here, significant speed-ups can be expected by prescreening of interfragment 2-electron integrals needed to evaluate the ECI Hamiltonian (see eq 46). In particular, for distant fragments, the NDDO approximation^17^ could be used to neglect many 2-electron integrals in the *J* terms and possibly entire *K* terms. For fragments in close proximity, many integrals contributing to the *J* terms will need to be evaluated, whereas only a few integrals contributing to the *K* terms will be required. Such integral prescreening would speed up both the integral evaluation as well as the contractions needed to evaluate the ECI Hamiltonian matrix elements (eqs 45 and 49). On a different note, for very large ECI expansions, large speed-ups could be achieved by employing a Davidson diagonalization.^62^

## 6 Conclusions

We have presented the ECI method for the calculation of electronic states of multichromophoric systems. It is based on an excitonic approach, i.e., it is essentially a variational calculation in the basis of antisymmetrized products of site states, but including not only GS and LE products but also MLE products. The Hamiltonian matrix is evaluated within McWeeny’s group function theory. We also present a systematic approach to include embedding via arbitrary point charges in the site-state calculations, either in a self-consistent way (EHF) or using point charges obtained from fitting the ESP; these are to be contrasted from the FDA. Overall, ECI has the desired properties listed in the introduction.

Testing the ECI method on the cases of **G**_**4**_ and **MgG_4_^2+^** systems leads to a number of conclusions. For a supramolecular (e.g., hydrogen-bonded) multichromophoric system, like **G**_**4**_, ECIS + FDA can be expected to yield qualitatively correct electronic states, with expected deviations of excitation energies around 0.1 eV. For the same systems, ECISD + FDA might reduce the deviations to below 0.1 eV, giving both qualitatively and quantitatively correct full-system states. Using either ESP or EHF atomic charges as the embedding charges in such systems is expected to yield spectra in almost perfect agreement with the ones obtained by direct quantum chemistry method, already within ECIS.

On the contrary, for systems with more strongly coupled chromophores (e.g., coordination metal complexes with organic ligands), ECI + FDA might potentially fail to reproduce the electronic spectrum even qualitatively, independent of the excitation rank (ECIS or ECISD). This is presumably due to the loss of size extensivity induced by nonzero couplings between the GS product and the LE products (i.e., violation of Brillouin’s theorem). Employing ESP embedding in such systems is expected to drastically improve the performance of ECI (over FDA), producing mostly all states qualitatively, but with excitation energies being off by 0.1–0.2 eV. Given that the site states with ESP embedding might not be sufficiently self-consistent for such systems, it is advisable to employ the larger ECISD expansion. Using EHF embedding in such strongly correlated multichromophoric systems showed qualitatively correct results, with average energy deviations below 0.1 eV, for both ECIS + EHF and ECISD + EHF methods. If the individual sites exhibit excited states of higher spin at low energies (e.g., low-lying triplets in systems with singlet ground state), then products of such states (e.g., triplet–triplet products as in singlet fission precursor states) can be expected to be present. In such situations, ECIS results should be cross-checked with ECISD computations, even if ECIS provides accurate spectra, as DLE configurations are dark.

In summary, the ECI method represents a general framework for the description of electronic states of multichromophoric systems. Employing the framework presented in this work, one could derive expressions for some other properties of the ECI states (e.g., spin–orbit couplings and wave function overlaps). Some other properties—especially gradients and nonadiabatic couplings vectors—would require additional theory development to deal with the required derivative terms (e.g., related to the embedding charges or the site-state density matrices^16,17^). Once ECI is extended by the mentioned properties, we envision that the method can be eventually employed for nonadiabatic dynamics.

## Appendix A

### Spin Adaptation of the ECI Basis

In the main text, we employed the compact site-state notation, e.g., *Fa*_*F*_, with *F* being the site index and *a*_*F*_ being a general index (or other identifier) for the site state. The compact notation is used, e.g., in the labels of the ESDs . In order to discuss the spin adaptation, it is more convenient to use an extended notation. In this case,  denotes a state of site *F* in multiplicity X (S for singlet, D for doublet, T for triplet, ...), *n*^*F*^ denotes the index of the site state in the manifold X (S_0_, S_1_, T_1_, ...), and *m*_*s*_^*F*^ is the corresponding spin projection. For example, 2T_3_^+1^ is the *m*_*s*_ = +1 component of the T_3_ state of fragment 2.

Let us denote a general ESD in the expanded notation as

28

First, we present the procedure for constructing the matrix of the  operator in the basis of the ESDs. The  operator of the full system can be written as

29

where  is the operator of the total spin of fragment *F*,  is the operator of its *z*-component, and  and  are the spin ladder operators of the corresponding fragments that change the *m*_*s*_^*F*^ or *m*_*s*_^*G*^ quantum numbers of the corresponding site states by ±1 with the corresponding prefactors, e.g., . The result of acting with  onto |Φ⟩ can then be obtained from standard angular momentum algebra and reads

30

where operators  and , respectively, increase and decrease the *m*_*s*_^*F*^ and *m*_*s*_^*G*^ values by 1 in the corresponding site states in |Φ⟩ (like operators  and , but without the prefactors). The factors *A* and *B* are

31

32

Using these equations, it is straightforward to obtain the **S**^2^ matrix elements in the basis of the ESDs, as within the strong orthogonality assumption, only  and  are nonzero.

Next, we illustrate the flow of the spin adaptation procedure of the ECISD basis for an exemplary two-chromophore system. Let us assume that a set of singlet and triplet site states has been given on each of the two fragments. This yields the initial (nonspin-adapted) ECISD basis containing the following ESDs

Here, the indices *i* and *j* independently run over all provided site states of fragments 1 and 2, respectively. The *m*_*s*_ value for the singlet site states are omitted since they are necessarily zero. Now, if we are interested in the singlet and triplet full-system states, we need to keep only ESDs with *M*_*S*_ = 0

for the description of the singlet manifold, and ESDs with *M*_*S*_ = 1

for the description of the triplet manifold.

After the **S**^2^ matrix is built in the basis of the ESDs with *M*_*S*_ = 0 and diagonalized, one obtains two singlet ECSFs (in both compact and expanded notation)

33

34

The other linear combination of the six *M*_*S*_ = 0 ESDs produces triplet and quintet ESCFs, which are discarded. The ESCFs used for the computation of the triplet states are obtained from the spin adaptation of the ESDs with *M*_*S*_ = 1

35

36

37

The final ECISD basis for the singlet manifold (to be used in the construction of the excitonic Hamiltonian matrix) contains all ESDs contributing to the singlet ECSFs, which are the four ESDs seen in eqs 33 and 34. The ESDs |1S_*i*_,2T_*j*_^0^⟩ and |1T_*i*_^0^,2S_*j*_⟩ are not needed to compute the full-system singlet states.

We note that the spin adaptation procedure is fully general, i.e., it would yield the minimal set of ESDs needed to span the states of any *S*, for any provided set of site states. Nevertheless, if the site states of too few different *s*^*F*^-values are provided, it might be impossible to construct ECSFs of any desired *S* (e.g., if one provides only singlet site states on each fragment, one cannot calculate full-system triplet states).

As a second example for the spin adaptation procedure, here we show the ECSF definitions of the TT-DLE and CT ECSFs of **MgG_4_^2+^** at geometry **R**_1_, as given in Table 4. These two ECSFs are given by

38

and

39

where fragment indices 1^+^ and 5^+^ denote that those fragments are in a certain state with the different charge than the site states used in ECI. Fragment 1 is guanine which is in all ECI configurations neutral, while here it acts as the CT donor and hence has charge 1+. On the other hand, fragment 5 is Mg^2+^, and in the CT product, it has charge 1+ since it acts as the CT acceptor.

## Appendix B

### Evaluation of ECI Integrals

The three types of integrals appearing in the ECI formulas for the full-system Hamiltonian matrix elements (eqs 8–10 modified by eq 18) are

40

41

42

Note that we omit the indices of the site states in the symbols for the densities and density matrices, as the evaluation of the integrals does not depend on the involved state/transition densities/density matrices. The *V* and *J* integrals are calculated by expressing the density matrices in the basis of the products of atomic orbitals (AOs) used in the site-state calculations

43

where  are coefficients of the density matrix in the AO product basis. Hence, the *V* and *J* integrals simplify to

44

45

where

46

are one- (nuclear–electron attraction) and two-electron integrals in the AO basis.

The *K* integrals are less straightforward, as they do not simplify to expressions containing full (either state or transition) density matrices when integrated over spin. Namely, we can write

47

where  are partial density matrices. Their coefficients in the MO product basis can be calculated from the wave functions of the site states as

48

where annihilation and creation operators without bar operate only on alpha spin orbitals, while the ones with bar operate only on beta spin orbitals. The density matrix coefficients can then easily be rotated to the AO product basis via the MO coefficients.  and  are actually alpha and beta densities and are (in principle) nonzero for all the state densities and for transition densities between the states with the same *m*_*s*_ values, while they are zero for transition densities between the states with different *m*_*s*_ values. On the other hand,  and  are nonzero only for transition densities between the site states with Δ*m*_*s*_ = ± 1, respectively. Finally, after integrating over ω_1_ and ω_2_ and expressing the partial densities in the basis of AO products, the *K* integral simplifies to

49

regardless of the *s* and *m*_*s*_ values of all (nominally four: *a*_*F*_, *b*_*F*_, *a*_*G*_, and *b*_*G*_) site states involved in the *K* term. Also, note that the two-electron integrals here are not the same as the ones needed for the *J* integrals (for *K*, two AOs with the same coordinate are coming from two different fragments).

The site-state “overlap” integrals in eq 21 are also calculated from the partial density matrix coefficients as

50

where the *D̃* terms are the AO product coefficients of the partial density matrices of the site states for which the “overlap” integral is calculated, while *S*_*il*_^*FG*^ and *S*_*jk*_^*FG*^ are overlaps of the AOs from different fragments. Note that there are no  and  as they are zero for the state densities.

Gaussian 16 provides total state density matrices for all states and αα and ββ transition density matrices between ground state and all excited states (even triplets with *m*_*s*_ = 0). Thus, there are several classes of missing densities. First, partial αα and ββ transition densities between two excited states, both having *m*_*s*_ = 0 (e.g. X_*n*_^0^ → Y_*m*_^0^), are obtained from the corresponding ground-to-excited transition densities as^54^

51

where σ = α, β, **D**s are the corresponding matrices of the density matrix coefficient in the AO product basis, and **S** is the AO overlap matrix. This formula is exact for CIS states, while for other types, one can construct the excited-to-excited densities directly from the CI coefficients. Regarding the nonzero βα partial densities, they can be calculated using the Wigner–Eckart theorem^36−38^ as

52

53

54

55

where *A* and *B* are Wigner 3*j* symbols and *m*′ can be any value (not dependent of *m*), as long as it does not yield *B* = 0. The same formula can be used to calculate the spin difference density **Q**, if some βα density is known. The **Q** density for a certain (pair of) state(s) can also be generated from **Q** of a corresponding (pair of) state(s) with different *m* value as

56

57

58

For example, in this work, we calculated

59

The corresponding nonzero αβ densities can be obtained as transpose of the matching βα ones. For example

60

The only densities that could not be calculated by the Wigner–Eckart theorem with a given set of densities printed by Gaussian in this work are αα and ββ state densities of the triplet states with *m* = ± 1. These are hence calculated from the CI coefficients by eq 48. We would like to emphasize that our implementation of density generation is fully general. Given a list of requested densities, it considers the densities that are printed by the quantum chemistry program, adds the ones it can calculate by eq 51 from the read ones (if the usage of the formula is enabled by the user), and only then adds the ones that need to be calculated from the explicit CI coefficients. In this way, every density is obtained in the cheapest way, where reading from the quantum chemistry program is cheapest, and calculating from the CI coefficients is most expensive. Each time densities are added, the algorithm also adds all densities it can generate from the present ones by the Wigner–Eckart theorem. The procedure is stopped after all requested densities have been generated. The schematic display of the described procedure for all density matrices used in this work is shown on Figure S2.

The required one- and two-electron integrals (eq 46)—as well as the AO overlap integrals needed in eq 50—were calculated using the PySCF Python package,^63,64^ in particular with the libcint library.^65^ In order to calculate the *V*, *J*, *K*, and *O* integrals using the above equations, the density matrix coefficients and the one- and two-electron integrals have to be given in the same basis set. However, the ordering and normalization conventions of the basis functions differ between Gaussian and PySCF. Therefore, after extracting and computing all the density matrix coefficients from Gaussian’s output, we transform them to match the PySCF convention. The AO integrals computed by PySCF were then used without any modification.

## Supporting Information Available

Full notation of the ECI Hamiltonian; energies and oscillator strengths of guanine excited site states in the **G**_**4**_ and **MgG_4_^2+^** systems with three types of embedding charges; dominant electronic configurations contributing to the guanine excited site states; corresponding hole/particle orbitals; ECI coefficients and ECI diagnostics of the full-system singlet states on symmetric geometries of **G**_**4**_ and **MgG_4_^2+^** systems calculated with six considered ECI variants and FEM; excitation energies and oscillator strengths of **MgG_4_^2+^** on the symmetric geometry calculated with the FEM, ECIS, and ECISD methods applying the “real” FDA (embedding charges of Mg^2+^ set to zero); characters of the direct CIS full-system states for **G**_**4**_ and **MgG_4_^2+^**; and scheme for construction of the all density matrices from the Gaussian’s output (PDF)
Supporting Python code of the SHARC-ECI and SHARC-GAUSSIAN interfaces, ECI module, and all their dependencies; example input files for ECI calculations using HF and CIS with Gaussian (ZIP)
Cartesian coordinates of the S_0_-optimized structures of **G**_**4**_ and **MgG_4_^2+^** and of the Wigner-sampled structures used in Figures 5 and 6. Molden files with the results of the frequency calculations for the S_0_ optimized structures of **G**_**4**_ and **MgG_4_^2+^** (ZIP)

## List of Acronyms

AO: atomic orbital
CT: charge transfer (product, state)
DLE: double-local excitation (product)
ECI: excitonic configuration interaction
ECSF: excitonic configuration-state function
EHF: excitonic Hartree–Fock (method, charges, embedding)
ESD: excitonic Slater determinant
ESP: electrostatic potential (charges, embedding)
FDA: frozen density approximation
FEM: Frenkel exciton model
GFT: group function theory
GS: ground state (product)
LE: local excitation (product)
MAD: mean absolute deviation
MLE: multilocal excitation (product)
MSD: mean signed deviation
RESP: restrained electrostatic potential (fit, charges)
RMSD: root-mean-squared deviation
TT-DLE: triplet–triplet double-local excitation (product, state)
