# Supplementary material for: Excitonic Configuration Interaction: Going Beyond the Frenkel Exciton Model
Source: J Chem Theory Comput. 2024 Jun 17;20(13):5609–34. doi: 10.1021/acs.jctc.4c00157 (PMC11238547; doi:10.1021/acs.jctc.4c00157)
Supplement: Supplementary file 1 — ct4c00157_si_001.pdf [file ct4c00157_si_001.pdf]

Supporting Information: Excitonic Configuration Interaction: Going Beyond the Frenkel Exciton Model

Tomislav Piteša,[*,†] Severin Polonius,[†,‡] Leticia González,[*,†] and Sebastian Mai[*,†]

†*Institute of Theoretical Chemistry, Faculty of Chemistry, University of Vienna, A-1090 Vienna, Austria*

‡*University of Vienna, Vienna Doctoral School in Chemistry (DoSChem), Währinger Str. 42, 1090 Vienna, Austria.*

E-mail: tomislav.pitesa@univie.ac.at; leticia.gonzalez@univie.ac.at; sebastian.mai@univie.ac.at

S1 Full notation of the ECI Hamiltonian

In this section, we summarize the full ECI Hamiltonian for the readers. The entire ECI Hamiltonian is given by Equation (20) in the main manuscript, repeated here for convenience:

$$\begin{aligned}\hat{H}^{\mathrm{ECI}} &= \hat{H}_{\mathrm{GS-GS}} + \hat{H}_{\mathrm{LE-LE}} + \hat{H}_{\mathrm{DLE-DLE}} + \dots \\ &+ \hat{H}_{\mathrm{GS-LE}} + \hat{H}_{\mathrm{GS-DLE}} + \hat{H}_{\mathrm{LE-DLE}} + \dots \\ &+ \text{adjoint}.\end{aligned} \tag{S1}$$

We note that this ECI Hamiltonian looks the same regardless of the employed embedding, except for the modifications in Equation (18). In the case of EHF embedding, some terms will tend towards zero, e.g., $\hat{H}_{\mathrm{GS-LE}}$ due to the excitonic version of Brillouin's theorem.

With $\widetilde{Y}^{FG}_{a_F,b_F|a_G,b_G} = \widetilde{J}^{FG}_{a_F,b_F|a_G,b_G} - \widetilde{K}^{FG}_{a_F,b_F|a_G,b_G}$, the $\hat{H}_{\mathrm{GS-GS}}$ term in the ECI Hamiltonian in manuscript reads

$$\hat{H}_{\mathrm{GS-GS}} = \left|\widetilde{\mathbf{0}}\right\rangle \sum_F \left[\widetilde{\mathcal{E}}^F_0 + \frac{1}{2}\sum_{G\neq F} \widetilde{Y}^{FG}_{0,0|0,0}\right] \left\langle\widetilde{\mathbf{0}}\right|. \tag{S2}$$

It contains not only the sum of site state energies, but also an interaction term for each pair of fragments.

The $\hat{H}_{\mathrm{LE-LE}}$ term is conveniently divided according to the number of the differing site states (noted in the superscript) as:

$$\hat{H}_{\mathrm{LE-LE}} = \hat{H}^{(0)}_{\mathrm{LE-LE}} + \hat{H}^{(1)}_{\mathrm{LE-LE}} + \hat{H}^{(2)}_{\mathrm{LE-LE}}, \tag{S3}$$

$$\hat{H}^{(0)}_{\mathrm{LE-LE}} = \sum_F \sum_{a_F>0} \left|\widetilde{\mathbf{0}}^{Fa_F}\right\rangle \left[\widetilde{\mathcal{E}}^F_{a_F} + \sum_{G\neq F}\left(\widetilde{\mathcal{E}}^G_0 + \widetilde{Y}^{FG}_{a_F,a_F|0,0} + \frac{1}{2}\sum_{H\neq F,G} \widetilde{Y}^{GH}_{0,0|0,0}\right)\right] \left\langle\widetilde{\mathbf{0}}^{Fa_F}\right|, \tag{S4}$$

$$\hat{H}^{(1)}_{\mathrm{LE-LE}} = \sum_F \sum_{a_F>0} \sum_{\substack{b_F>0 \\ b_F\neq a_F}} \left|\widetilde{\mathbf{0}}^{Fb_F}\right\rangle \sum_{G\neq F} \widetilde{Y}^{FG}_{b_F,a_F|0,0} \left\langle\widetilde{\mathbf{0}}^{Fa_F}\right|, \tag{S5}$$

$$\hat{H}^{(2)}_{\mathrm{LE-LE}} = \sum_F \sum_{a_F>0} \sum_{G\neq F} \sum_{a_G>0} \left|\widetilde{\mathbf{0}}^{Fa_F}\right\rangle \widetilde{Y}^{FG}_{a_F,0|0,a_G} \left\langle\widetilde{\mathbf{0}}^{Ga_G}\right|. \tag{S6}$$

The $\hat{H}^{(0)}_{\mathrm{LE-LE}}$ gives rise to the diagonal matrix elements of all LE products, $\hat{H}^{(1)}_{\mathrm{LE-LE}}$ forms the coupling between two LEs on the same fragment, while $\hat{H}^{(2)}_{\mathrm{LE-LE}}$ gives the couplings between LEs on two different sites.

The DLE–DLE term is also conveniently separated in the same way as

$$\hat{H}_{\mathrm{DLE-DLE}} = \hat{H}^{(0)}_{\mathrm{DLE-DLE}} + \hat{H}^{(1)}_{\mathrm{DLE-DLE}} + \hat{H}^{(2)}_{\mathrm{DLE-DLE}} \tag{S7}$$

$$\begin{aligned}\hat{H}^{(0)}_{\mathrm{DLE-DLE}} = \frac{1}{2}\sum_{F}\sum_{a_F>0}\sum_{G\neq F}\sum_{a_G>0}\left|\widetilde{\mathbf{0}}^{Fa_F,Ga_G}\right\rangle \Bigg[\widetilde{\mathcal{E}}^{F}_{a_F} + \widetilde{\mathcal{E}}^{G}_{a_G} + \widetilde{Y}^{FG}_{a_F,a_F|a_G,a_G} \\ + \sum_{H\neq F,G}\left(\widetilde{\mathcal{E}}^{H}_{0} + \widetilde{Y}^{FH}_{a_F,a_F|0,0} + \widetilde{Y}^{GH}_{a_G,a_G|0,0} + \frac{1}{2}\sum_{I\neq F,G,H}\widetilde{Y}^{HI}_{0,0|0,0}\right)\Bigg]\left\langle\widetilde{\mathbf{0}}^{Fa_F,Ga_G}\right|\end{aligned} \tag{S8}$$

$$\hat{H}^{(1)}_{\mathrm{DLE-DLE}} = \sum_{F}\sum_{a_F>0}\sum_{G\neq F}\sum_{a_G>0}\sum_{\substack{b_G>0\\ b_G\neq a_G}}\left|\widetilde{\mathbf{0}}^{Fa_F,Gb_G}\right\rangle\left[\widetilde{Y}^{FG}_{a_F,a_F|b_G,a_G} + \sum_{H\neq F,G}\widetilde{Y}^{HG}_{0,0|b_G,a_G}\right]\left\langle\widetilde{\mathbf{0}}^{Fa_F,Ga_G}\right| \tag{S9}$$

$$\begin{aligned}\hat{H}^{(2)}_{\mathrm{DLE-DLE}} = \frac{1}{2}\sum_{F}\sum_{a_F>0}\sum_{\substack{b_F>0\\ b_F\neq a_F}}\sum_{G\neq F}\sum_{a_G>0}\sum_{\substack{b_G>0\\ b_G\neq a_G}}\left|\widetilde{\mathbf{0}}^{Fb_F,Gb_G}\right\rangle\widetilde{Y}^{FG}_{b_F,a_F|b_G,a_G}\left\langle\widetilde{\mathbf{0}}^{Fa_F,Ga_G}\right| \\ + \sum_{F}\sum_{a_F>0}\sum_{G\neq F}\sum_{a_G>0}\sum_{H\neq F,G}\sum_{a_H>0}\left|\widetilde{\mathbf{0}}^{Fa_F,Ga_G}\right\rangle\widetilde{Y}^{GH}_{a_G,0|0,a_H}\left\langle\widetilde{\mathbf{0}}^{Fa_F,Ha_H}\right|\end{aligned} \tag{S10}$$

The $\hat{H}^{(0)}_{\mathrm{DLE-DLE}}$ again gives rise to the diagonal matrix elements of DLEs. The $\hat{H}^{(1)}_{\mathrm{DLE-DLE}}$ couples pairs of DLEs, where the same two fragments are excited and where one fragment is in the same site state and the other fragment in a different site state between bra and ket. The $\hat{H}^{(2)}_{\mathrm{DLE-DLE}}$ couples two types of DLEs—(i) ones with the same two excited fragments, but both in different excited site states (first term), and (ii) ones with a common fragment excited in the same excited site state while the other excited fragments are different (G and H) (second term).

The $\hat{H}_{\mathrm{GS-LE}}$ and $\hat{H}_{\mathrm{GS-DLE}}$ terms cover the couplings the excitonic ground state and LEs or DLEs. As the ground state differs by one or two site states, respectively, from LEs or

DLEs, these read as

$$\hat{H}_{\mathrm{GS-LE}} = \sum_{F} \sum_{a_F>0} \left|\widetilde{\mathbf{0}}\right\rangle \sum_{G\neq F} \widetilde{Y}^{FG}_{0,a_F|0,0} \left\langle \widetilde{\mathbf{0}}^{Fa_F}\right|, \tag{S11}$$

$$\hat{H}_{\mathrm{GS-DLE}} = \frac{1}{2} \sum_{F} \sum_{a_F>0} \sum_{G\neq F} \sum_{a_G>0} \left|\widetilde{\mathbf{0}}\right\rangle \widetilde{Y}^{FG}_{0,a_F|0,a_G} \left\langle \widetilde{\mathbf{0}}^{Fa_F,Ga_G}\right|. \tag{S12}$$

On the contrary, the $\hat{H}_{\mathrm{LE-DLE}}$ term covers the coupling between ESDs differing in one or two site states and hence can be separated again as:

$$\hat{H}_{\mathrm{LE-DLE}} = \hat{H}^{(1)}_{\mathrm{LE-DLE}} + \hat{H}^{(2)}_{\mathrm{LE-DLE}}, \tag{S13}$$

$$\hat{H}^{(1)}_{\mathrm{LE-DLE}} = \sum_{F} \sum_{a_F>0} \sum_{G\neq F} \sum_{a_G>0} \left|\widetilde{\mathbf{0}}^{Fa_F}\right\rangle \left[\widetilde{Y}^{FG}_{a_F,a_F|0,a_G} + \sum_{H\neq F,G} \widetilde{Y}^{GH}_{0,a_G|0,0} \right] \left\langle \widetilde{\mathbf{0}}^{Fa_F,Ga_G}\right|, \tag{S14}$$

$$\hat{H}^{(2)}_{\mathrm{LE-DLE}} = \sum_{F} \sum_{a_F>0} \sum_{\substack{b_F>0\\ b_F\neq a_F}} \sum_{G\neq F} \sum_{a_G>0} \left|\widetilde{\mathbf{0}}^{Fb_F}\right\rangle \widetilde{Y}^{FG}_{b_F,a_F|0,a_G} \left\langle \widetilde{\mathbf{0}}^{Fa_F,Ga_G}\right|. \tag{S15}$$

For comparison, the Frenkel Hamiltonian can be written in this notation as

$$\begin{aligned}
\hat{H}^{\mathrm{FEM}} = \hat{H}_{\mathrm{GS-GS}} &- \frac{1}{2} \left|\widetilde{\mathbf{0}}\right\rangle \sum_{F} \sum_{G\neq F} \widetilde{Y}^{FG}_{0,0|0,0} \left\langle \widetilde{\mathbf{0}}\right| \\
&+ \hat{H}^{(0)}_{\mathrm{LE-LE}} - \sum_{F} \sum_{a_F>0} \left|\widetilde{\mathbf{0}}^{Fa_F}\right\rangle \sum_{G\neq F} \left[\widetilde{Y}^{FG}_{a_F,a_F|0,0} + \frac{1}{2} \sum_{H\neq F,G} \widetilde{Y}^{GH}_{0,0|0,0} \right] \left\langle \widetilde{\mathbf{0}}^{Fa_F}\right| \\
&+ \hat{H}^{(2)}_{\mathrm{LE-LE}},
\end{aligned} \tag{S16}$$

which is equivalent to equation (11) in the main text.

S2 Site state energetics and characters

Table S1: Excitation energies of guanine site states and oscillator strengths of the singlet site states on the Frank–Condon geometries of $\mathbf{G}_4$ and $\mathbf{MgG}_4^{2+}$ systems with three different embedding schemes.

	— $\mathbf{G}_4$ —			— $\mathbf{MgG}_4^{2+}$ —		
State	FDA	ESP	EHF	FDA	ESP	EHF
— excitation energies (eV) —						
T_1	4.4236	4.4392	4.4101	4.2636	3.8891	4.0589
T_2	4.5262	4.6532	4.7129	4.4645	5.0476	5.0260
T_3	5.4344	5.6352	5.6788	5.3186	5.4362	5.5319
S_1	6.3595	6.4814	6.4966	6.2388	6.0573	6.1932
S_2	6.8571	7.1291	7.1083	6.6164	7.1489	7.1664
S_3	7.2063	7.2568	7.3879	7.0870	7.7501	7.9336
S_4	7.7325	7.8722	7.9164	7.6387	8.3283	8.4319
S_5	7.7974	8.3651	8.3746	7.7838	8.4741	8.5326
S_6	8.3170	8.4633	8.4774	8.2969	8.7817	8.8313
S_7	8.5128	8.7220	8.7550	8.4190	8.8108	8.8463
— oscillator strengths —						
S_1	0.3172	0.2924	0.2813	0.2964	0.2765	0.2693
S_2	0.0007	0.5793	0.5916	0.0006	0.4678	0.5301
S_3	0.5478	0.0006	0.0007	0.5841	0.0106	0.0121
S_4	0.0109	0.0102	0.0100	0.0107	0.5437	0.2616
S_5	0.0010	0.0848	0.0866	0.0010	0.0106	0.0145
S_6	0.0725	0.0011	0.0011	0.0745	0.0114	0.5430
S_7	0.0016	0.0010	0.0005	0.0026	0.0072	0.0883

Table S2: Expansion of each site state labeled on Figure 2 in manuscript, in the terms of MO excitations or in the terms of already defined site states, divided per $\pi\pi^*$, $n\pi^*$ and πRy character. Notation Hn (Ln) refers to the n-th canonical MO below (above) HOMO (LUMO). All MOs needed to construct all site states are shown on Figure S1.

Label	Expansion	Label	Expansion	Label	Expansion
$\pi\pi_1^*$	$\lvert \mathrm{H} \rightarrow \mathrm{L1} \rangle$	$n\pi_1^*$	$\lvert n_\mathrm{O} \rightarrow \pi^*_\mathrm{CO} \rangle$	$\pi\mathrm{Ry}_6$	$\lvert \mathrm{H} \rightarrow \mathrm{L} \rangle$
$\pi\pi_2^*$	$\lvert \mathrm{H} \rightarrow \mathrm{L2} \rangle$	$n\pi_2^*$	$\lvert n_\mathrm{N7} \rightarrow \mathrm{L2} \rangle$	$\pi\mathrm{Ry}_5$	$\lvert \mathrm{H} \rightarrow \mathrm{L3} \rangle$
$\pi\pi_3^*$	$\frac{1}{\sqrt{2}}\left(\pi\pi_1^* - \pi\pi_2^*\right)$	$n\pi_3^*$	$\frac{1}{\sqrt{2}}\left(n\pi_1^* - n\pi_2^*\right)$		
$\pi\pi_4^*$	$\frac{1}{\sqrt{2}}\left(\pi\pi_1^* + \pi\pi_2^*\right)$	$n\pi_4^*$	$\frac{1}{\sqrt{2}}\left(n\pi_1^* + n\pi_2^*\right)$		
$\pi\pi_5^*$	$\lvert \mathrm{H} \rightarrow \mathrm{L5} \rangle$	$n\pi_5^*$	$\lvert n_\mathrm{N3} \rightarrow \mathrm{L1} \rangle$		
$\pi\pi_6^*$	$\frac{1}{\sqrt{2}}\left(\pi\pi_5^* + \lvert \mathrm{H1} \rightarrow \mathrm{L1} \rangle\right)$				
$\pi\pi_7^*$	$\frac{1}{\sqrt{2}}\left(\lvert \mathrm{H1} \rightarrow \mathrm{L2} \rangle + \lvert \mathrm{H3} \rightarrow \mathrm{L2} \rangle\right)$				

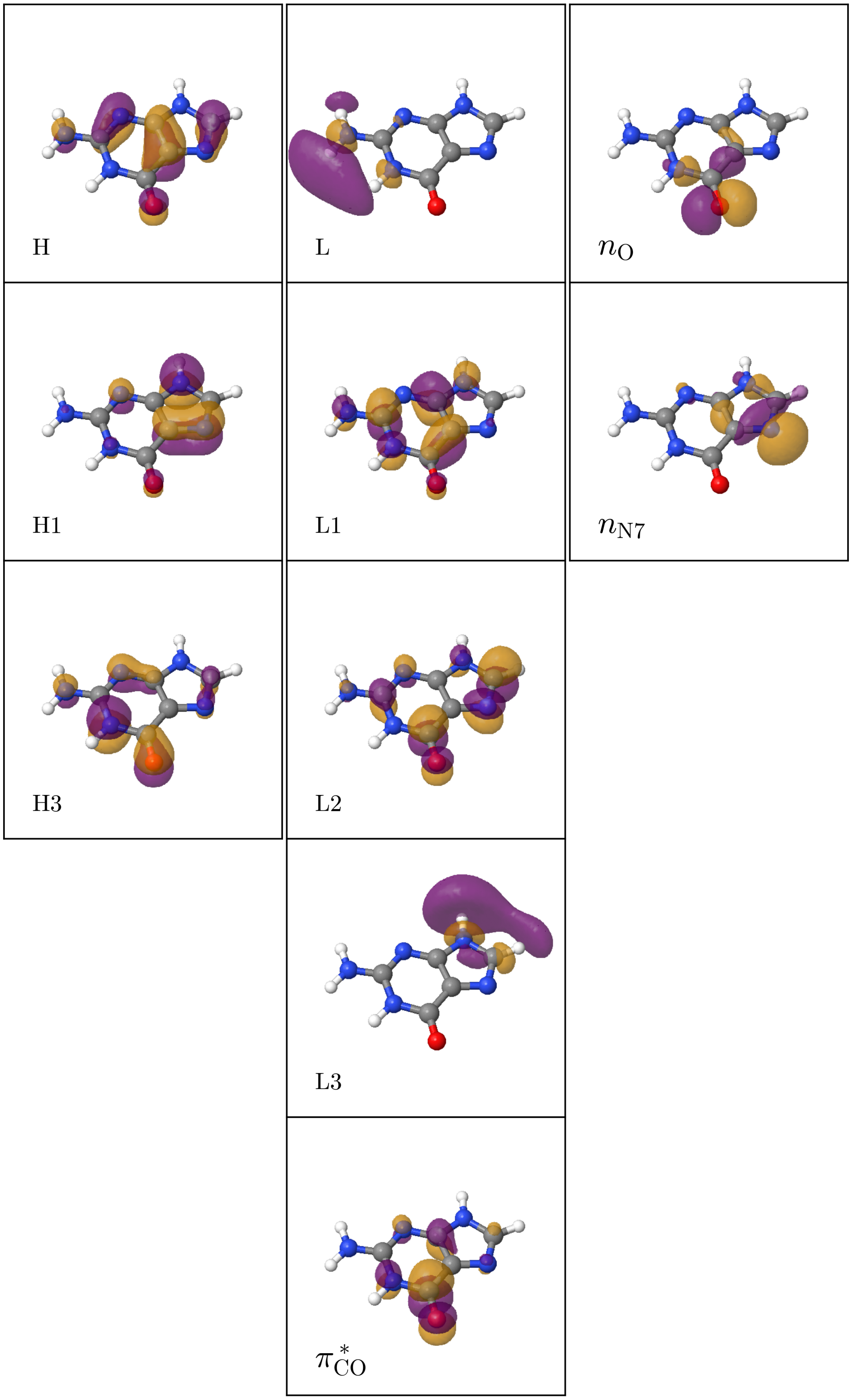

Figure S1: Molecular orbitals of guanine on FC geometry of $\mathbf{G}_4$ needed to descripe guanine site states in both $\mathbf{G}_4$ and $\mathbf{MgG}_4^{2+}$ systems with all three embedding schemes.

S3 Excitonic CI coefficients for G_4

S3.1 G_4 – FEM

Table S3: ECI wave functions of the first 9 singlet states of $\mathbf{G}_4$, calculated with FEM. Only the ECSFs having the squared value of the ECI coefficient in any of considered states greater than 0.005 are shown.

	States								
ECSFs	S_0	S_1	S_2	S_3	S_4	S_5	S_6	S_7	S_8
$^1\widetilde{\mathbf{0}}$	1.000	0.000	−0.000	−0.000	0.000	0.000	0.000	0.000	−0.000
$^1\widetilde{\mathbf{0}}^{3S_1}$	0.000	0.500	−0.706	−0.035	0.500	−0.000	−0.000	−0.000	−0.000
$^1\widetilde{\mathbf{0}}^{4S_1}$	0.000	0.500	0.035	−0.706	−0.500	−0.000	0.000	−0.000	−0.000
$^1\widetilde{\mathbf{0}}^{2S_1}$	0.000	0.500	−0.035	0.706	−0.500	0.000	−0.000	−0.000	0.000
$^1\widetilde{\mathbf{0}}^{1S_1}$	0.000	0.500	0.706	0.035	0.500	0.000	0.000	0.000	0.000
$^1\widetilde{\mathbf{0}}^{3S_2}$	0.000	0.000	−0.000	0.000	−0.000	0.706	0.041	−0.500	−0.500
$^1\widetilde{\mathbf{0}}^{1S_2}$	0.000	0.000	−0.000	−0.000	0.000	0.706	0.041	0.500	0.500
$^1\widetilde{\mathbf{0}}^{2S_2}$	0.000	0.000	−0.000	0.000	0.000	−0.041	0.706	0.500	−0.500
$^1\widetilde{\mathbf{0}}^{4S_2}$	0.000	0.000	0.000	−0.000	0.000	−0.041	0.706	−0.500	0.500
PR	1.000	4.002	2.018	2.018	4.002	2.013	2.013	4.000	4.000
E_0	1.000	0.000	0.000	0.000	0.000	0.000	0.000	0.000	0.000
E_1	0.000	1.000	1.000	1.000	1.000	1.000	1.000	1.000	1.000
E_2	0.000	0.000	0.000	0.000	0.000	0.000	0.000	0.000	0.000

S3.2 G_4 – FDA

Table S4: ECI wave functions of the first 9 singlet states of $\mathbf{G}_4$, calculated with ECIS method without embedding. Only the ECSFs having the squared value of the ECI coefficient in any of considered states greater than 0.005 are shown.

	States								
	S_0	S_1	S_2	S_3	S_4	S_5	S_6	S_7	S_8
ECSFs $^1\tilde{\mathbf{0}}$	0.995	−0.101	−0.000	−0.000	−0.000	0.000	0.000	0.000	−0.006
$^1\tilde{\mathbf{0}}^{2S_1}$	0.050	0.489	−0.684	0.003	−0.479	−0.130	0.161	−0.033	−0.079
$^1\tilde{\mathbf{0}}^{1S_1}$	0.050	0.489	−0.003	−0.684	0.479	0.130	−0.033	−0.161	−0.079
$^1\tilde{\mathbf{0}}^{3S_1}$	0.050	0.489	0.003	0.684	0.479	0.130	0.033	0.161	−0.079
$^1\tilde{\mathbf{0}}^{4S_1}$	0.050	0.489	0.684	−0.003	−0.479	−0.130	−0.161	0.033	−0.079
$^1\tilde{\mathbf{0}}^{2S_3}$	0.011	0.085	−0.169	0.038	−0.137	0.476	−0.678	−0.015	0.484
$^1\tilde{\mathbf{0}}^{1S_3}$	0.011	0.085	−0.038	−0.169	0.137	−0.476	−0.015	0.678	0.484
$^1\tilde{\mathbf{0}}^{3S_3}$	0.011	0.085	0.038	0.169	0.137	−0.476	0.015	−0.678	0.484
$^1\tilde{\mathbf{0}}^{4S_3}$	0.011	0.085	0.169	−0.038	−0.137	0.476	0.678	0.015	0.484
$^1\tilde{\mathbf{0}}^{2S_6}$	0.002	−0.027	0.045	0.001	0.037	0.078	−0.114	0.008	0.099
$^1\tilde{\mathbf{0}}^{1S_6}$	0.002	−0.027	−0.001	0.045	−0.037	−0.078	0.008	0.114	0.099
$^1\tilde{\mathbf{0}}^{3S_6}$	0.002	−0.027	0.001	−0.045	−0.037	−0.078	−0.008	−0.114	0.099
$^1\tilde{\mathbf{0}}^{4S_6}$	0.002	−0.027	−0.045	−0.001	0.037	0.078	0.114	−0.008	0.099
PR	1.021	4.352	2.275	2.275	4.703	4.824	2.358	2.358	4.553
E_0	0.990	0.010	0.000	0.000	0.000	0.000	0.000	0.000	0.000
E_1	0.010	0.990	1.000	1.000	1.000	1.000	1.000	1.000	1.000
E_2	0.000	0.000	0.000	0.000	0.000	0.000	0.000	0.000	0.000

Table S5: ECI wave functions of the first 9 singlet states of $\mathbf{G}_4$, calculated with ECISD method without embedding. Only the ECSFs having the squared value of the ECI coefficient in any of considered states greater than 0.005 are shown.

	States								
ECSFs	S_0	S_1	S_2	S_3	S_4	S_5	S_6	S_7	S_8
$^1\widetilde{\mathbf{0}}$	0.995	−0.103	0.000	0.000	−0.000	0.000	0.000	0.000	−0.005
$^1\widetilde{\mathbf{0}}^{1S_1}$	0.050	0.485	0.681	−0.018	−0.477	−0.132	0.162	0.040	−0.083
$^1\widetilde{\mathbf{0}}^{3S_1}$	0.050	0.485	−0.681	0.018	−0.477	−0.132	−0.162	−0.040	−0.083
$^1\widetilde{\mathbf{0}}^{2S_1}$	0.050	0.485	0.018	0.681	0.477	0.132	0.040	−0.162	−0.083
$^1\widetilde{\mathbf{0}}^{4S_1}$	0.050	0.485	−0.018	−0.681	0.477	0.132	−0.040	0.162	−0.083
$^1\widetilde{\mathbf{0}}^{1S_3}$	0.011	0.088	0.173	0.034	−0.139	0.473	−0.674	−0.011	0.480
$^1\widetilde{\mathbf{0}}^{3S_3}$	0.011	0.088	−0.173	−0.034	−0.139	0.473	0.674	0.011	0.480
$^1\widetilde{\mathbf{0}}^{2S_3}$	0.011	0.088	−0.034	0.173	0.139	−0.473	−0.011	0.674	0.480
$^1\widetilde{\mathbf{0}}^{4S_3}$	0.011	0.088	0.034	−0.173	0.139	−0.473	0.011	−0.674	0.480
$^1\widetilde{\mathbf{0}}^{1S_6}$	0.002	−0.026	−0.045	0.003	0.037	0.081	−0.118	−0.012	0.102
$^1\widetilde{\mathbf{0}}^{3S_6}$	0.002	−0.026	0.045	−0.003	0.037	0.081	0.118	0.012	0.102
$^1\widetilde{\mathbf{0}}^{4S_6}$	0.002	−0.026	0.003	0.045	−0.037	−0.081	0.012	−0.118	0.102
$^1\widetilde{\mathbf{0}}^{2S_6}$	0.002	−0.026	−0.003	−0.045	−0.037	−0.081	−0.012	0.118	0.102
PR	1.022	4.503	2.313	2.313	4.781	4.953	2.418	2.418	4.682
E_0	0.989	0.011	0.000	0.000	0.000	0.000	0.000	0.000	0.000
E_1	0.011	0.975	0.995	0.995	0.995	0.991	0.992	0.992	0.992
E_2	0.000	0.014	0.005	0.005	0.005	0.009	0.008	0.008	0.008

S3.3 $\mathbf{G}_4$ – ESP embedding

Table S6: ECI wave functions of the first 9 singlet states of $\mathbf{G}_4$, calculated with ECIS method with ESP-embedding. Only the ECSFs having the squared value of the ECI coefficient in any of considered states greater than 0.005 are shown.

	States								
ECSFs	S_0	S_1	S_2	S_3	S_4	S_5	S_6	S_7	S_8
$^1\widetilde{\mathbf{0}}$	1.000	0.024	−0.000	−0.000	0.000	−0.000	0.000	0.000	−0.009
$^1\widetilde{\mathbf{0}}^{1S_1}$	−0.012	0.500	−0.553	−0.434	−0.495	−0.067	0.035	0.066	0.007
$^1\widetilde{\mathbf{0}}^{2S_1}$	−0.012	0.500	−0.434	0.553	0.495	0.067	−0.066	0.035	0.007
$^1\widetilde{\mathbf{0}}^{4S_1}$	−0.012	0.500	0.434	−0.553	0.495	0.067	0.066	−0.035	0.007
$^1\widetilde{\mathbf{0}}^{3S_1}$	−0.012	0.500	0.553	0.434	−0.495	−0.067	−0.035	−0.066	0.007
$^1\widetilde{\mathbf{0}}^{3S_2}$	0.005	−0.007	−0.074	−0.009	0.067	−0.496	0.043	−0.702	0.499
$^1\widetilde{\mathbf{0}}^{1S_2}$	0.005	−0.007	0.074	0.009	0.067	−0.496	−0.043	0.702	0.499
$^1\widetilde{\mathbf{0}}^{2S_2}$	0.005	−0.007	0.009	−0.074	−0.067	0.496	−0.702	−0.043	0.499
$^1\widetilde{\mathbf{0}}^{4S_2}$	0.005	−0.007	−0.009	0.074	−0.067	0.496	0.702	0.043	0.499
PR	1.001	4.006	3.872	3.872	4.154	4.146	2.062	2.062	4.020
E_0	0.999	0.001	0.000	0.000	0.000	0.000	0.000	0.000	0.000
E_1	0.001	0.999	1.000	1.000	1.000	1.000	1.000	1.000	1.000
E_2	0.000	0.000	0.000	0.000	0.000	0.000	0.000	0.000	0.000

Table S7: ECI wave functions of the first 9 singlet states of $\mathbf{G}_4$, calculated with ECISD method with ESP-embedding. Only the ECSFs having the squared value of the ECI coefficient in any of considered states greater than 0.005 are shown.

	States								
ECSFs	S_0	S_1	S_2	S_3	S_4	S_5	S_6	S_7	S_8
$^1\widetilde{\mathbf{0}}$	1.000	0.024	0.000	−0.000	0.000	0.000	0.000	0.000	−0.009
$^1\widetilde{\mathbf{0}}^{2S_1}$	−0.012	0.500	−0.643	−0.284	0.495	0.067	0.039	0.063	0.008
$^1\widetilde{\mathbf{0}}^{3S_1}$	−0.012	0.500	−0.284	0.643	−0.495	−0.067	−0.063	0.039	0.008
$^1\widetilde{\mathbf{0}}^{4S_1}$	−0.012	0.500	0.643	0.284	0.495	0.067	−0.039	−0.063	0.008
$^1\widetilde{\mathbf{0}}^{1S_1}$	−0.012	0.500	0.284	−0.643	−0.495	−0.067	0.063	−0.039	0.008
$^1\widetilde{\mathbf{0}}^{2S_2}$	0.005	−0.008	0.074	−0.010	−0.067	0.495	0.003	0.703	0.499
$^1\widetilde{\mathbf{0}}^{4S_2}$	0.005	−0.008	−0.074	0.010	−0.067	0.495	−0.003	−0.703	0.499
$^1\widetilde{\mathbf{0}}^{1S_2}$	0.005	−0.008	0.010	0.074	0.067	−0.495	0.703	−0.003	0.499
$^1\widetilde{\mathbf{0}}^{3S_2}$	0.005	−0.008	−0.010	−0.074	0.067	−0.495	−0.703	0.003	0.499
PR	1.002	4.013	2.820	2.820	4.157	4.153	2.050	2.050	4.028
E_0	0.999	0.001	0.000	0.000	0.000	0.000	0.000	0.000	0.000
E_1	0.001	0.999	1.000	1.000	1.000	0.999	0.999	0.999	0.999
E_2	0.000	0.001	0.000	0.000	0.000	0.001	0.001	0.001	0.001

S3.4 $\mathbf{G}_4$ – EHF embedding

Table S8: ECI wave functions of the first 9 singlet states of $\mathbf{G}_4$, calculated with ECIS method with EHF-embedding. Only the ECSFs having the squared value of the ECI coefficient in any of considered states greater than 0.005 are shown.

	States								
ECSFs	S_0	S_1	S_2	S_3	S_4	S_5	S_6	S_7	S_8
$^1\widetilde{\mathbf{0}}$	1.000	−0.000	0.000	0.000	0.000	0.000	−0.000	0.000	0.005
$^1\widetilde{\mathbf{0}}^{4S_1}$	0.000	0.499	−0.445	0.548	0.499	0.032	0.010	−0.040	0.027
$^1\widetilde{\mathbf{0}}^{3S_1}$	0.000	0.499	−0.548	−0.445	−0.499	−0.032	0.040	0.010	0.027
$^1\widetilde{\mathbf{0}}^{1S_1}$	0.000	0.499	0.548	0.445	−0.499	−0.032	−0.040	−0.010	0.027
$^1\widetilde{\mathbf{0}}^{2S_1}$	0.000	0.499	0.445	−0.548	0.499	0.032	−0.010	0.040	0.027
$^1\widetilde{\mathbf{0}}^{1S_2}$	−0.002	−0.028	0.035	−0.022	−0.032	0.499	0.392	−0.587	0.499
$^1\widetilde{\mathbf{0}}^{2S_2}$	−0.002	−0.028	−0.022	−0.035	0.032	−0.499	−0.587	−0.392	0.499
$^1\widetilde{\mathbf{0}}^{4S_2}$	−0.002	−0.028	0.022	0.035	0.032	−0.499	0.587	0.392	0.499
$^1\widetilde{\mathbf{0}}^{3S_2}$	−0.002	−0.028	−0.035	0.022	−0.032	0.499	−0.392	0.587	0.499
PR	1.000	4.028	3.866	3.866	4.036	4.033	3.514	3.514	4.029
E_0	1.000	0.000	0.000	0.000	0.000	0.000	0.000	0.000	0.000
E_1	0.000	1.000	1.000	1.000	1.000	1.000	1.000	1.000	1.000
E_2	0.000	0.000	0.000	0.000	0.000	0.000	0.000	0.000	0.000

Table S9: ECI wave functions of the first 9 singlet states of $\mathbf{G}_4$, calculated with ECISD method with EHF-embedding. Only the ECSFs having the squared value of the ECI coefficient in any of considered states greater than 0.005 are shown.

	States								
ECSFs	S_0	S_1	S_2	S_3	S_4	S_5	S_6	S_7	S_8
$^1\widetilde{\mathbf{0}}$	1.000	−0.000	0.000	−0.000	0.000	0.000	−0.000	−0.000	0.005
$^1\widetilde{\mathbf{0}}^{4S_1}$	0.000	0.499	−0.584	0.396	−0.499	−0.032	−0.039	−0.013	0.027
$^1\widetilde{\mathbf{0}}^{1S_1}$	0.000	0.499	−0.396	−0.584	0.499	0.032	−0.013	0.039	0.027
$^1\widetilde{\mathbf{0}}^{2S_1}$	0.000	0.499	0.584	−0.396	−0.499	−0.032	0.039	0.013	0.027
$^1\widetilde{\mathbf{0}}^{3S_1}$	0.000	0.499	0.396	0.584	0.499	0.032	0.013	−0.039	0.027
$^1\widetilde{\mathbf{0}}^{4S_2}$	−0.002	−0.028	−0.033	−0.025	−0.032	0.499	0.438	−0.554	0.499
$^1\widetilde{\mathbf{0}}^{3S_2}$	−0.002	−0.028	−0.025	0.033	0.032	−0.499	0.554	0.438	0.499
$^1\widetilde{\mathbf{0}}^{1S_2}$	−0.002	−0.028	0.025	−0.033	0.032	−0.499	−0.554	−0.438	0.499
$^1\widetilde{\mathbf{0}}^{2S_2}$	−0.002	−0.028	0.033	0.025	−0.032	0.499	−0.438	0.554	0.499
PR	1.000	4.028	3.544	3.544	4.036	4.033	3.825	3.825	4.029
E_0	1.000	0.000	0.000	0.000	0.000	0.000	0.000	0.000	0.000
E_1	0.000	1.000	1.000	1.000	1.000	1.000	1.000	1.000	1.000
E_2	0.000	0.000	0.000	0.000	0.000	0.000	0.000	0.000	0.000

S4 Characters of direct CIS states for G_4

Table S10: Excitation energies (eV), oscillator strengths, guanine-centered (GC) and guanine-to-guanine charge-transfer (GGCT) percentage of the character of the first 28 excited singlet states of $\mathbf{G}_4$ on symmetric geometry.

State	E_{exc}	f_{osc}	GC	GGCT
S_1	6.403	0.000	0.997	0.003
S_2	6.488	0.744	0.998	0.002
S_3	6.488	0.744	0.998	0.002
S_4	6.526	0.000	0.998	0.002
S_5	7.040	0.000	0.997	0.003
S_6	7.069	1.253	0.997	0.003
S_7	7.069	1.253	0.997	0.003
S_8	7.153	0.000	0.998	0.002
S_9	7.441	0.000	0.984	0.016
S_{10}	7.441	0.000	0.984	0.016
S_{11}	7.441	0.000	0.984	0.016
S_{12}	7.441	0.003	0.984	0.016
S_{13}	7.920	0.000	0.992	0.008
S_{14}	7.921	0.000	0.992	0.008
S_{15}	7.921	0.000	0.992	0.008
S_{16}	7.921	0.035	0.992	0.008
S_{17}	8.340	0.000	0.996	0.004
S_{18}	8.354	0.204	0.996	0.004
S_{19}	8.354	0.204	0.996	0.004
S_{20}	8.376	0.000	0.996	0.004
S_{21}	8.473	0.000	0.994	0.006
S_{22}	8.473	0.000	0.994	0.006
S_{23}	8.473	0.000	0.994	0.006
S_{24}	8.473	0.004	0.994	0.006
S_{25}	8.767	0.000	0.998	0.002
S_{26}	8.767	0.000	0.998	0.002
S_{27}	8.767	0.001	0.998	0.002
S_{28}	8.767	0.000	0.998	0.002

S5 Performance of FEM and ECI with "real" FDA on MgG_4^{2+} system

Table S11: Excitation energies (and oscillator strengths from the ground state for singlets) of the first eight singlet and first eight triplet excited states of $\mathbf{MgG_4^{2+}}$, calculated with the direct full-system method, the FEM, ECIS+FDA and ECISD+FDA, where in the FEM and ECI calculations the "real" FDA was employed, i.e., not even the +2 charge of Mg^{2+} was present in the embedding charges.

State	Direct	FEM	ECIS	ECISD
	— Triplet excitation energies (eV) —			
T_1	4.0030	4.2636	5.4516	4.4241
T_2	4.0031	4.2636	5.4516	4.4241
T_3	4.0031	4.2636	5.4516	4.4241
T_4	4.0033	4.2636	5.4516	4.4241
T_5	5.0502	4.4644	6.3039	5.2505
T_6	5.0505	4.4645	6.3039	5.2505
T_7	5.0505	4.4645	6.3039	5.2505
T_8	5.0508	4.4646	6.3040	5.2506
	— Singlet excitation energies (eV) —			
S_1	5.9465	6.2193	7.4478	6.7448
S_2	6.0134	6.2301	7.4967	6.7937
S_3	6.0134	6.2301	7.4967	6.7937
S_4	6.2041	6.2444	8.5322	7.1060
S_5	7.0940	6.6155	9.0486	8.0159
S_6	7.1255	6.6163	9.0486	8.0159
S_7	7.1255	6.6163	9.1020	8.0576
S_8	7.1664	6.6175	9.3300	8.0641
	— Singlet oscillator strenghts (eV) —			
S_1	0.0103	0.0000	0.0045	0.0001
S_2	0.6627	0.5170	0.7337	0.6103
S_3	0.6627	0.5170	0.7337	0.6103
S_4	0.0000	0.0621	0.0000	0.0000
S_5	0.0000	0.0000	1.1407	1.1042
S_6	0.9413	0.0009	1.1407	1.1042
S_7	0.9413	0.0009	0.4177	0.3860
S_8	0.2926	0.0009	0.0000	0.0000

S6 Excitonic CI coefficients for MgG_4^{2+}

S6.1 MgG_4^{2+} – FEM

Table S12: ECI wave functions of the first 9 singlet states of $\mathbf{MgG_4^{2+}}$, calculated with FEM. Only the ECSFs having the squared value of the ECI coefficient in any of considered states greater than 0.005 are shown.

	States								
ECSFs	S_0	S_1	S_2	S_3	S_4	S_5	S_6	S_7	S_8
$^1\widetilde{\mathbf{0}}$	1.000	0.000	0.000	0.000	0.000	−0.000	0.000	−0.000	−0.000
$^1\widetilde{\mathbf{0}}^{1S_1}$	0.000	0.500	0.081	−0.702	0.498	−0.034	−0.006	0.012	0.011
$^1\widetilde{\mathbf{0}}^{4S_1}$	0.000	−0.500	0.702	0.081	0.498	−0.034	−0.012	−0.006	−0.011
$^1\widetilde{\mathbf{0}}^{2S_1}$	0.000	−0.500	−0.702	−0.081	0.498	−0.034	0.012	0.006	−0.011
$^1\widetilde{\mathbf{0}}^{3S_1}$	0.000	0.500	−0.081	0.702	0.498	−0.034	0.006	−0.012	0.011
$^1\widetilde{\mathbf{0}}^{4S_3}$	0.000	0.012	−0.006	−0.012	0.032	0.498	−0.063	−0.703	−0.499
$^1\widetilde{\mathbf{0}}^{1S_3}$	0.000	−0.012	−0.012	0.006	0.032	0.498	−0.703	0.063	0.499
$^1\widetilde{\mathbf{0}}^{3S_3}$	0.000	−0.012	0.012	−0.006	0.032	0.498	0.703	−0.063	0.499
$^1\widetilde{\mathbf{0}}^{2S_3}$	0.000	0.012	0.006	0.012	0.032	0.498	0.063	0.703	−0.499
PR	1.000	4.016	2.056	2.056	4.075	4.070	2.048	2.048	4.045
E_0	1.000	0.000	0.000	0.000	0.000	0.000	0.000	0.000	0.000
E_1	0.000	1.000	1.000	1.000	1.000	1.000	1.000	1.000	1.000
E_2	0.000	0.000	0.000	0.000	0.000	0.000	0.000	0.000	0.000

S6.2 MgG_4^{2+} – FDA

Table S13: ECI wave functions of the first 9 singlet states of $\mathbf{MgG_4^{2+}}$, calculated with ECIS method without embedding. Only the ECSFs having the squared value of the ECI coefficient in any of considered states greater than 0.005 are shown.

ECSFs	S_0	S_1	S_2	S_3	S_4	S_5	S_6	S_7	S_8
$^1\widetilde{\mathbf{0}}$	0.970	0.000	−0.000	−0.000	0.093	−0.000	−0.000	−0.000	−0.145
$^1\widetilde{\mathbf{0}}^{3S_1}$	−0.082	0.486	−0.657	−0.186	0.431	0.023	−0.168	0.102	−0.237
$^1\widetilde{\mathbf{0}}^{4S_1}$	−0.082	−0.486	0.186	−0.657	0.431	−0.168	−0.023	−0.102	−0.237
$^1\widetilde{\mathbf{0}}^{2S_1}$	−0.082	−0.486	−0.186	0.657	0.431	0.168	0.023	−0.102	−0.237
$^1\widetilde{\mathbf{0}}^{1S_1}$	−0.082	0.486	0.657	0.186	0.431	−0.023	0.168	0.102	−0.237
$^1\widetilde{\mathbf{0}}^{1S_5}$	−0.082	0.042	0.040	0.002	−0.054	0.008	0.119	0.063	−0.007
$^1\widetilde{\mathbf{0}}^{4S_5}$	−0.082	−0.042	0.002	−0.040	−0.054	−0.119	0.008	−0.063	−0.007
$^1\widetilde{\mathbf{0}}^{3S_5}$	−0.082	0.042	−0.040	−0.002	−0.054	−0.008	−0.119	0.063	−0.007
$^1\widetilde{\mathbf{0}}^{2S_5}$	−0.082	−0.042	−0.002	0.040	−0.054	0.119	−0.008	−0.063	−0.007
$^1\widetilde{\mathbf{0}}^{3S_3}$	0.034	0.108	−0.167	−0.063	0.243	−0.021	0.675	−0.485	0.434
$^1\widetilde{\mathbf{0}}^{4S_3}$	0.034	−0.108	0.063	−0.167	0.243	0.675	0.021	0.485	0.434
$^1\widetilde{\mathbf{0}}^{2S_3}$	0.034	−0.108	−0.063	0.167	0.243	−0.675	−0.021	0.485	0.434
$^1\widetilde{\mathbf{0}}^{1S_3}$	0.034	0.108	0.167	0.063	0.243	0.021	−0.675	−0.485	0.434
PR	1.129	4.457	2.653	2.653	6.569	2.400	2.400	4.502	6.475
E_0	0.941	0.000	0.000	0.000	0.009	0.000	0.000	0.000	0.021
E_1	0.059	1.000	1.000	1.000	0.991	1.000	1.000	1.000	0.979
E_2	0.000	0.000	0.000	0.000	0.000	0.000	0.000	0.000	0.000

(Column group header: States)

Table S14: ECI wave functions of the first 9 singlet states of $\mathbf{MgG}_4^{2+}$, calculated with ECISD method without embedding. Only the ECSFs having the squared value of the ECI coefficient in any of considered states greater than 0.005 are shown.

	States								
ECSFs	S_0	S_1	S_2	S_3	S_4	S_5	S_6	S_7	S_8
$^1\widetilde{\mathbf{0}}$	0.967	−0.000	−0.000	0.000	0.120	−0.000	−0.000	−0.153	−0.000
$^1\widetilde{\mathbf{0}}^{1S_5}$	−0.085	0.032	0.025	−0.011	−0.029	0.012	0.092	−0.056	−0.046
$^1\widetilde{\mathbf{0}}^{3S_5}$	−0.085	0.032	−0.025	0.011	−0.029	−0.012	−0.092	−0.056	−0.046
$^1\widetilde{\mathbf{0}}^{4S_5}$	−0.085	−0.032	0.011	0.025	−0.029	−0.092	0.012	−0.056	0.046
$^1\widetilde{\mathbf{0}}^{2S_5}$	−0.085	−0.032	−0.011	−0.025	−0.029	0.092	−0.012	−0.056	0.046
$^1\widetilde{\mathbf{0}}^{4S_1}$	−0.084	−0.476	0.429	0.512	0.443	−0.174	−0.015	−0.169	0.108
$^1\widetilde{\mathbf{0}}^{3S_1}$	−0.084	0.476	−0.512	0.429	0.443	0.015	−0.174	−0.169	−0.108
$^1\widetilde{\mathbf{0}}^{1S_1}$	−0.084	0.476	0.512	−0.429	0.443	−0.015	0.174	−0.169	−0.108
$^1\widetilde{\mathbf{0}}^{2S_1}$	−0.084	−0.476	−0.429	−0.512	0.443	0.174	0.015	−0.169	0.108
$^1\widetilde{\mathbf{0}}^{3S_3}$	0.036	0.110	−0.129	0.125	0.186	−0.003	0.662	0.442	0.474
$^1\widetilde{\mathbf{0}}^{4S_3}$	0.036	−0.110	0.125	0.129	0.186	0.662	0.003	0.442	−0.474
$^1\widetilde{\mathbf{0}}^{2S_3}$	0.036	−0.110	−0.125	−0.129	0.186	−0.662	−0.003	0.442	−0.474
$^1\widetilde{\mathbf{0}}^{1S_3}$	0.036	0.110	0.129	−0.125	0.186	0.003	−0.662	0.442	0.474
$^1\widetilde{\mathbf{0}}^{2S_1,4S_1}$	0.005	0.085	0.000	−0.000	−0.079	0.000	0.000	0.033	−0.022
$^1\widetilde{\mathbf{0}}^{1S_1,3S_1}$	0.005	−0.085	−0.000	0.000	−0.079	0.000	0.000	0.033	0.022
$^1\widetilde{\mathbf{0}}^{2S_1,3S_1}$	0.003	−0.000	0.087	0.008	−0.083	−0.022	0.018	0.038	−0.000
$^1\widetilde{\mathbf{0}}^{1S_1,4S_1}$	0.003	−0.000	−0.087	−0.008	−0.083	0.022	−0.018	0.038	−0.000
$^1\widetilde{\mathbf{0}}^{3S_1,4S_1}$	0.003	0.000	0.008	−0.087	−0.083	0.018	0.022	0.038	0.000
$^1\widetilde{\mathbf{0}}^{1S_1,2S_1}$	0.003	0.000	−0.008	0.087	−0.083	−0.018	−0.022	0.038	0.000
PR	1.143	4.861	4.859	4.859	6.295	2.595	2.595	6.376	4.933
E_0	0.935	0.000	0.000	0.000	0.014	0.000	0.000	0.023	0.000
E_1	0.064	0.959	0.957	0.957	0.926	0.956	0.956	0.911	0.955
E_2	0.001	0.041	0.043	0.043	0.060	0.044	0.044	0.066	0.045

S6.3 MgG_4^{2+} – ESP embedding

Table S15: ECI wave functions of the first 9 singlet states of $\mathbf{MgG_4^{2+}}$, calculated with ECIS method with ESP-embedding. Only the ECSFs having the squared value of the ECI coefficient in any of considered states greater than 0.005 are shown.

	States								
ECSFs	S_0	S_1	S_2	S_3	S_4	S_5	S_6	S_7	S_8
$^{1}\widetilde{\mathbf{0}}$	0.994	−0.000	−0.000	−0.000	0.072	−0.000	−0.000	−0.056	−0.000
$^{1}\widetilde{\mathbf{0}}^{3S_1}$	−0.044	0.498	0.000	−0.702	0.478	0.014	−0.080	−0.135	−0.042
$^{1}\widetilde{\mathbf{0}}^{2S_1}$	−0.044	−0.498	−0.702	−0.000	0.478	0.080	0.014	−0.135	0.042
$^{1}\widetilde{\mathbf{0}}^{4S_1}$	−0.044	−0.498	0.702	0.000	0.478	−0.080	−0.014	−0.135	0.042
$^{1}\widetilde{\mathbf{0}}^{1S_1}$	−0.044	0.498	−0.000	0.702	0.478	−0.014	0.080	−0.135	−0.042
$^{1}\widetilde{\mathbf{0}}^{4S_2}$	0.017	−0.041	0.080	0.014	0.136	0.702	0.002	0.481	−0.498
$^{1}\widetilde{\mathbf{0}}^{2S_2}$	0.017	−0.041	−0.080	−0.014	0.136	−0.702	−0.002	0.481	−0.498
$^{1}\widetilde{\mathbf{0}}^{1S_2}$	0.017	0.041	−0.014	0.080	0.136	0.002	−0.702	0.481	0.498
$^{1}\widetilde{\mathbf{0}}^{3S_2}$	0.017	0.041	0.014	−0.080	0.136	−0.002	0.702	0.481	0.498
PR	1.024	4.063	2.055	2.055	4.742	2.058	2.058	4.658	4.077
E_0	0.988	0.000	0.000	0.000	0.005	0.000	0.000	0.003	0.000
E_1	0.012	1.000	1.000	1.000	0.995	1.000	1.000	0.997	1.000
E_2	0.000	0.000	0.000	0.000	0.000	0.000	0.000	0.000	0.000

Table S16: ECI wave functions of the first 9 singlet states of $\mathbf{MgG_4^{2+}}$, calculated with ECISD method with ESP-embedding. Only the ECSFs having the squared value of the ECI coefficient in any of considered states greater than 0.005 are shown.

	States								
ECSFs	S_0	S_1	S_2	S_3	S_4	S_5	S_6	S_7	S_8
$^{1}\widetilde{\mathbf{0}}$	0.994	−0.000	0.000	−0.000	0.073	−0.053	−0.000	−0.000	−0.000
$^{1}\widetilde{\mathbf{0}}^{3S_1}$	−0.044	0.496	0.006	−0.700	0.480	−0.110	−0.014	0.077	−0.040
$^{1}\widetilde{\mathbf{0}}^{4S_1}$	−0.044	−0.496	−0.700	−0.006	0.480	−0.110	0.077	0.014	0.040
$^{1}\widetilde{\mathbf{0}}^{2S_1}$	−0.044	−0.496	0.700	0.006	0.480	−0.110	−0.077	−0.014	0.040
$^{1}\widetilde{\mathbf{0}}^{1S_1}$	−0.044	0.496	−0.006	0.700	0.480	−0.110	0.014	−0.077	−0.040
$^{1}\widetilde{\mathbf{0}}^{2S_2}$	0.017	−0.040	0.077	−0.012	0.114	0.483	0.699	0.010	−0.495
$^{1}\widetilde{\mathbf{0}}^{4S_2}$	0.017	−0.040	−0.077	0.012	0.114	0.483	−0.699	−0.010	−0.495
$^{1}\widetilde{\mathbf{0}}^{1S_2}$	0.017	0.040	0.012	0.077	0.114	0.483	−0.010	0.699	0.495
$^{1}\widetilde{\mathbf{0}}^{3S_2}$	0.017	0.040	−0.012	−0.077	0.114	0.483	0.010	−0.699	0.495
PR	1.025	4.122	2.086	2.086	4.675	4.564	2.092	2.092	4.150
E_0	0.988	0.000	0.000	0.000	0.005	0.003	0.000	0.000	0.000
E_1	0.012	0.992	0.992	0.992	0.979	0.984	0.991	0.991	0.991
E_2	0.000	0.008	0.008	0.008	0.016	0.013	0.009	0.009	0.009

S6.4 MgG_4^{2+} – EHF embedding

Table S17: ECI wave functions of the first 9 singlet states of $\mathbf{MgG_4^{2+}}$, calculated with ECIS method with EHF-embedding. Only the ECSFs having the squared value of the ECI coefficient in any of considered states greater than 0.005 are shown.

	States								
ECSFs	S_0	S_1	S_2	S_3	S_4	S_5	S_6	S_7	S_8
$^1\tilde{\mathbf{0}}$	1.000	0.000	0.000	−0.000	−0.008	0.006	0.000	0.000	0.000
$^1\tilde{\mathbf{0}}^{3S_1}$	0.005	0.499	−0.005	−0.707	0.497	−0.053	0.006	−0.017	0.022
$^1\tilde{\mathbf{0}}^{4S_1}$	0.005	−0.499	−0.707	0.005	0.497	−0.053	−0.017	−0.006	−0.022
$^1\tilde{\mathbf{0}}^{2S_1}$	0.005	−0.499	0.707	−0.005	0.497	−0.053	0.017	0.006	−0.022
$^1\tilde{\mathbf{0}}^{1S_1}$	0.005	0.499	0.005	0.707	0.497	−0.053	−0.006	0.017	0.022
$^1\tilde{\mathbf{0}}^{4S_2}$	−0.003	0.023	0.013	0.012	0.052	0.497	−0.332	−0.624	−0.499
$^1\tilde{\mathbf{0}}^{3S_2}$	−0.003	−0.023	−0.012	0.013	0.052	0.497	0.624	−0.332	0.499
$^1\tilde{\mathbf{0}}^{1S_2}$	−0.003	−0.023	0.012	−0.013	0.052	0.497	−0.624	0.332	0.499
$^1\tilde{\mathbf{0}}^{2S_2}$	−0.003	0.023	−0.013	−0.012	0.052	0.497	0.332	0.624	−0.499
PR	1.000	4.023	2.003	2.003	4.113	4.110	3.056	3.056	4.033
E_0	1.000	0.000	0.000	0.000	0.000	0.000	0.000	0.000	0.000
E_1	0.000	1.000	1.000	1.000	1.000	1.000	1.000	1.000	1.000
E_2	0.000	0.000	0.000	0.000	0.000	0.000	0.000	0.000	0.000

Table S18: ECI wave functions of the first 9 singlet states of $\mathbf{MgG_4^{2+}}$, calculated with ECISD method with EHF-embedding. Only the ECSFs having the squared value of the ECI coefficient in any of considered states greater than 0.005 are shown.

	States								
ECSFs	S_0	S_1	S_2	S_3	S_4	S_5	S_6	S_7	S_8
$^1\tilde{\mathbf{0}}$	1.000	0.000	0.000	0.000	−0.008	0.006	0.000	0.000	−0.000
$^1\tilde{\mathbf{0}}^{4S_1}$	0.005	0.499	−0.706	−0.039	0.496	−0.052	0.006	0.017	−0.022
$^1\tilde{\mathbf{0}}^{3S_1}$	0.005	−0.499	0.039	−0.706	0.496	−0.052	0.017	−0.006	0.022
$^1\tilde{\mathbf{0}}^{1S_1}$	0.005	−0.499	−0.039	0.706	0.496	−0.052	−0.017	0.006	0.022
$^1\tilde{\mathbf{0}}^{2S_1}$	0.005	0.499	0.706	0.039	0.496	−0.052	−0.006	−0.017	−0.022
$^1\tilde{\mathbf{0}}^{3S_2}$	−0.003	0.023	−0.013	0.012	0.051	0.497	0.323	−0.628	0.499
$^1\tilde{\mathbf{0}}^{2S_2}$	−0.003	−0.023	−0.012	−0.013	0.051	0.497	−0.628	−0.323	−0.499
$^1\tilde{\mathbf{0}}^{4S_2}$	−0.003	−0.023	0.012	0.013	0.051	0.497	0.628	0.323	−0.499
$^1\tilde{\mathbf{0}}^{1S_2}$	−0.003	0.023	0.013	−0.012	0.051	0.497	−0.323	0.628	0.499
PR	1.001	4.024	2.016	2.016	4.115	4.112	3.000	3.000	4.037
E_0	0.999	0.000	0.000	0.000	0.000	0.000	0.000	0.000	0.000
E_1	0.000	1.000	1.000	1.000	1.000	1.000	1.000	1.000	1.000
E_2	0.000	0.000	0.000	0.000	0.000	0.000	0.000	0.000	0.000

S7 Characters of direct CIS states for MgG_4^{2+}

Table S19: Excitation energies (eV), oscillator strengths, guanine-centered (GC), guanine-to-guanine charge-transfer (GGCT) and guanine-to-metal charge-transfer (GMCT) percentage of the character of the first 28 excited singlet states of $\mathbf{MgG_4^{2+}}$ on symmetric geometry. The MC and MGCT percentages in all states are found to be negligible.

State	E_{exc}	f_{osc}	GC	GGCT	GMCT
S_1	5.947	0.010	0.944	0.008	0.042
S_2	6.013	0.663	0.945	0.007	0.042
S_3	6.013	0.663	0.945	0.007	0.042
S_4	6.204	0.000	0.958	0.007	0.031
S_5	7.094	0.000	0.969	0.006	0.021
S_6	7.126	0.941	0.972	0.007	0.018
S_7	7.126	0.941	0.972	0.007	0.018
S_8	7.166	0.292	0.974	0.007	0.016
S_9	7.705	0.000	0.138	0.065	0.793
S_{10}	7.716	0.004	0.140	0.065	0.787
S_{11}	7.716	0.004	0.140	0.065	0.787
S_{12}	7.756	0.008	0.146	0.064	0.779
S_{13}	7.869	0.004	0.961	0.024	0.014
S_{14}	7.869	0.004	0.961	0.024	0.014
S_{15}	7.870	0.028	0.957	0.024	0.018
S_{16}	7.871	0.000	0.963	0.024	0.012
S_{17}	8.215	0.000	0.955	0.022	0.021
S_{18}	8.322	0.439	0.869	0.047	0.080
S_{19}	8.322	0.439	0.869	0.047	0.080
S_{20}	8.382	0.057	0.703	0.105	0.189
S_{21}	8.496	0.014	0.916	0.040	0.041
S_{22}	8.497	0.000	0.862	0.062	0.073
S_{23}	8.502	0.016	0.970	0.018	0.009
S_{24}	8.502	0.016	0.970	0.018	0.009
S_{25}	8.528	0.000	0.267	0.301	0.425
S_{26}	8.530	0.194	0.209	0.328	0.457
S_{27}	8.530	0.194	0.209	0.328	0.457
S_{28}	8.573	0.056	0.395	0.255	0.346

Table S20: Excitation energies (eV), oscillator strengths, guanine-centered (GC), guanine-to-guanine charge-transfer (GGCT) and guanine-to-metal charge-transfer (GMCT) percentage of the character of the first 28 excited singlet states of $\mathbf{MgG}_4^{2+}$ on non-symmetric geometry $\mathbf{R}_1$. The MC and MGCT percentages in all states are found to be negligible.

State	E_{exc}	f_{osc}	GC	GGCT	GMCT
S_1	5.324	0.472	0.962	0.007	0.024
S_2	5.682	0.294	0.958	0.008	0.027
S_3	6.144	0.373	0.951	0.009	0.037
S_4	6.279	0.142	0.957	0.008	0.029
S_5	6.559	0.389	0.988	0.005	0.003
S_6	6.752	0.604	0.968	0.008	0.019
S_7	7.045	0.607	0.975	0.011	0.011
S_8	7.079	0.034	0.967	0.022	0.009
S_9	7.099	0.447	0.975	0.008	0.012
S_{10}	7.442	0.017	0.964	0.022	0.013
S_{11}	7.539	0.011	0.648	0.056	0.292
S_{12}	7.540	0.044	0.918	0.030	0.051
S_{13}	7.545	0.002	0.493	0.067	0.436
S_{14}	7.698	0.002	0.165	0.071	0.754
S_{15}	7.806	0.003	0.981	0.010	0.008
S_{16}	7.815	0.000	0.127	0.074	0.794
S_{17}	7.998	0.129	0.766	0.187	0.042
S_{18}	8.037	0.063	0.593	0.348	0.057
S_{19}	8.081	0.021	0.669	0.298	0.031
S_{20}	8.110	0.054	0.949	0.030	0.019
S_{21}	8.131	0.038	0.716	0.041	0.238
S_{22}	8.134	0.006	0.585	0.045	0.363
S_{23}	8.153	0.193	0.751	0.092	0.152
S_{24}	8.190	0.123	0.750	0.131	0.115
S_{25}	8.308	0.101	0.279	0.456	0.259
S_{26}	8.364	0.023	0.908	0.047	0.035
S_{27}	8.425	0.011	0.928	0.031	0.039
S_{28}	8.439	0.388	0.760	0.117	0.118

S8 Errors of the ECI calculations from the Section 5.5

Table S21: MADs (in eV) of excitation energies of the first 8 singlet excited states of $\mathbf{G}_4$ and $\mathbf{MgG}_4^{2+}$ for different ECI+EHF calculations and for different t_Q threshold for the EHF convergence, corresponding to Figure 8 in the manuscript.

System	$\log(t_Q)$	ECIS	ECISD	ECISDT	ECISDTQ
$\mathbf{G}_4$	0	0.0262	0.0238	0.0233	0.0233
	−1	0.0205	0.0211	0.0206	0.0206
	−2	0.0205	0.0211	0.0206	0.0206
	−3	0.0205	0.0211	0.0206	0.0206
	−4	0.0204	0.0210	0.0206	0.0206
$\mathbf{MgG}_4^{2+}$	0	0.1783	0.1269	0.1266	0.1266
	−1	0.0886	0.0902	0.0892	0.0892
	−2	0.0885	0.0898	0.0888	0.0888
	−3	0.0885	0.0898	0.0888	0.0888
	−4	0.0885	0.0898	0.0888	0.0888

S9 Construction of the density matrices

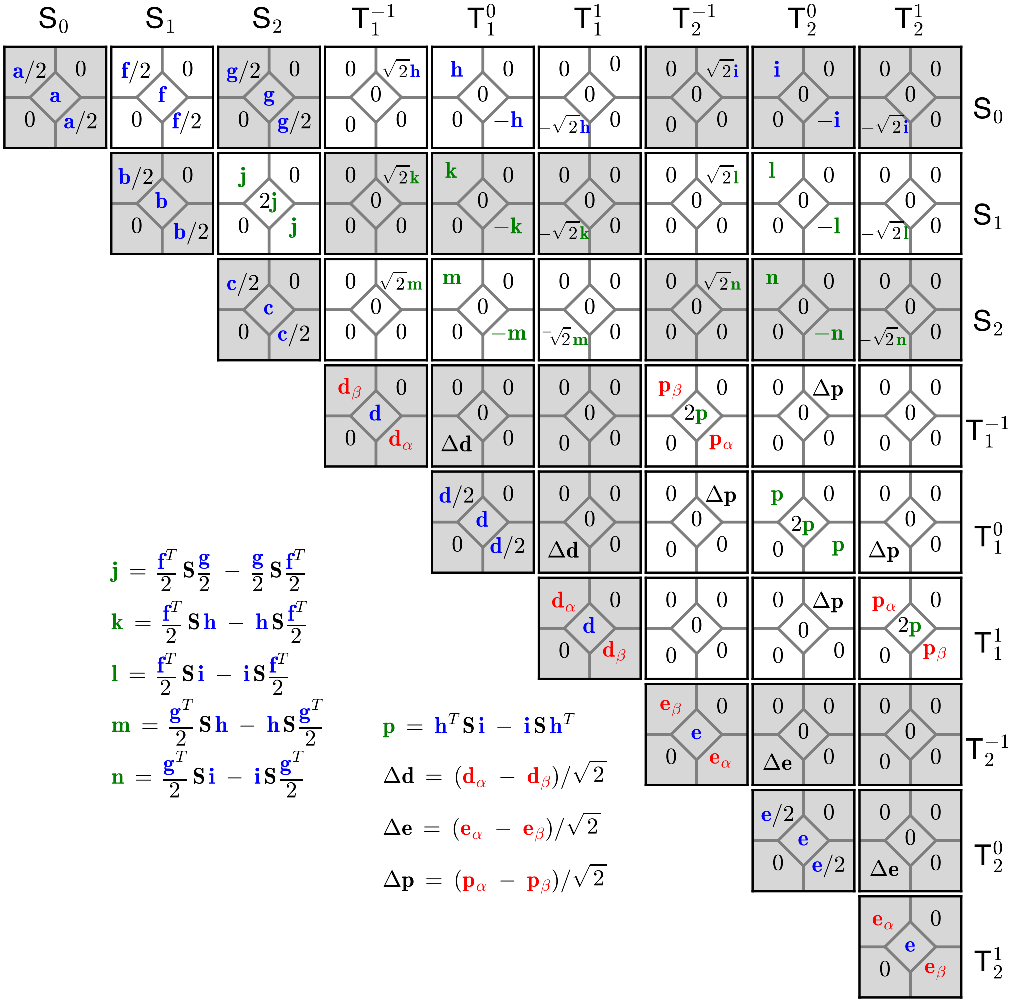

Figure S2: Schematic of how the density-matrix coefficients for different (pairs of) site states are obtained. For each row-column combination, a rhomb in the middle of the square represents the total density ($\widetilde{D}$). The four corners then correspond to $\widetilde{D}^{\alpha\alpha}$ (upper left), $\widetilde{D}^{\alpha\beta}$ (upper right), $\widetilde{D}^{\beta\alpha}$ (lower left) and $\widetilde{D}^{\beta\beta}$ (lower right). The fields are gray-white shaded to ease the distinguishing between different site states. The matrices of the density-matrix coefficients that are read directly from Gaussian's fchk file are coloured blue, the ones that are calculated by the Equation (51) are green, while those that are calculated from the CI and MO coefficients of the site states (read from Gaussian's rwf and fchk file, respectively) are coloured red. The **S** is the AO-overlap matrix. The density-matrix coefficients for the pairs of states in the lower triangle of the figure are obtained by transposing the matrices of the density-matrix coefficients from the upper triangle. While transposing, the spin labels also swap positions, e.g. $\widetilde{D}^{\alpha\beta}(\mathrm{T}_2^1, \mathrm{S}_1) = \widetilde{D}^{\beta\alpha}(\mathrm{S}_1, \mathrm{T}_2^1)^T$. Note that the figure can trivially be generalized from S_1, S_2, T_1, and T_2 to any pair of excited singlet/triplet site states.
